# Variation in urobiome composition over time in asymptomatic individuals with spinal cord injury and disease using intermittent catheterization

**DOI:** 10.1371/journal.pone.0356391

**Published:** 2026-10-30

**Authors:** Rochelle E. Tractenberg, Suzanne L. Groah, Ethan Newcomb, Mark Khemmani, Cara Joyce, Alan J. Wolfe, Christopher R. Riegner

**Affiliations:** 1 Collaborative for Research on Outcomes and –Metrics, Georgetown University, Washington, District of Columbia, United States of America; 2 Departments of Neurology, Rehabilitation Medicine, and Biostatistics, Bioinformatics and Biomathematics, Georgetown University, Washington, District of Columbia, United States of America; 3 MedStar National Rehabilitation Hospital, Washington, District of Columbia, United States of America; 4 MedStar Georgetown University Hospital, Washington, District of Columbia, United States of America; 5 Department of Microbiology and Immunology, Loyola University Chicago, Maywood, Illinois, United States of America; 6 Department of Medicine, Loyola University, Chicago, Illinois, United States of America; State Key Laboratory for Diagnosis and Treatment of Infectious Diseases, CHINA

## Abstract

Urinary tract infection (UTI) remains the most common infectious complication among individuals with neurogenic lower urinary tract dysfunction (NLUTD) due to spinal cord injury or disease (SCI/D). Despite widespread reliance on microbiological and symptom-based criteria for UTI diagnosis, significant ambiguity persists—especially in distinguishing clinically meaningful change from normal variability in urinary analysis results. This uncertainty contributes to overdiagnosis, inappropriate antibiotic use, and antimicrobial resistance. The present study sought to operationalize “normal variability” of the urinary microbiome (urobiome) among adults with NLUTD due to SCI/D. Using repeated samples collected from asymptomatic individuals over time, we analyzed inter- and intra-individual microbial composition to determine stability and fluctuation under baseline conditions. We observed wide intra- and inter-individual variability, substantial overlap between asymptomatic and pre-symptomatic states, and a consistent predominance of genera conventionally labeled as “uropathogens” including *Escherichia/Shigella, Klebsiella, Enterococcus, Staphylococcus*, and *Streptococcus,* even in the absence of symptoms. Bray-Curtis dissimilarities between paired samples ranged from 0 to 1, confirming substantial within-person compositional change over time. These findings suggest that assumptions drawn from cross-sectional studies—linking particular taxa or diversity values to health or disease—are not supported within individuals over time, at least in people with NLUTD due to SCI/D. Empirically characterizing normal within-person variability is a prerequisite both for interpreting urobiome results in this high-risk population and for improving the accuracy and stewardship of UTI diagnosis and treatment.

## Introduction

Accurate diagnosis of urinary tract infection (UTI) among individuals with neurogenic lower urinary tract dysfunction (NLUTD) due to spinal cord injury or disease (SCI/D) is a major clinical and scientific challenge [1]. Targeted quantitative PCR (qPCR) pathogen panels, expanded quantitative urine culture, and 16S rRNA gene sequencing have provided deeper resolution of microbial composition of what is now known to be a dynamic microbial ecosystem in the urinary tract, the urobiome [2]. These more advanced assays offer the potential to detect organisms missed by standard urine culture (SUC) and identify microbial or host-response signatures associated with disease risk [3]. The clinical utility of these approaches depends, however, on whether urobiome findings from cross-sectional studies reflect stable within-person characteristics — a question this study was designed to address.

Interest in 16S rRNA gene sequencing to characterize the urobiome and explore its potential diagnostic and prognostic relevance has been acute and increasing [4]. This work has fueled enthusiasm for defining “healthy” and “unhealthy” urobiome profiles [5]. However, often urobiome studies rely on cross-sectional samples and simplified summaries of community composition [6] that may obscure intra-individual temporal variability and/or may not capture intra-individual dynamics or reflect underlying biological states [2]. The range of normal within-person variability in 16S rRNA-based urobiome profiles is unknown; and group-level characterizations of “healthy” or “pathogenic” urobiome profiles have, to date, been derived from cross-sectional studies.

Cross-sectional approaches, although practical, impose several conceptual and methodological limitations. They treat the microbiome as a static entity, assuming that between-person differences observed at a single time point adequately represent stable biological features within individuals [7]. This assumption is especially tenuous in the urinary tract, where microbial abundance and composition can fluctuate rapidly in response to hydration, catheterization, antimicrobial exposure, and hormonal or immune factors [2,8]. Without first characterizing the range of normal within-person variability, it is not possible to determine whether a given microbial profile represents a meaningful deviation or simply a transient state captured at a single moment.

Nevertheless, studies have characterized entire groups by the predominance category most common among their members [9] even when a substantial proportion of cases share the same predominance profile [10]. This majority-rule summarization collapses a multidimensional and temporally variable microbial landscape into dichotomous group categories, erasing within-group heterogeneity and between-group overlap alike. The result is an inferential slide from observed group-level prevalence differences to claims about biological normality or pathology—a conclusion that cross-sectional data, which have never established what is normal within any individual, cannot support.

Methodologically, group-level comparisons of relative abundance or diversity indices obscure the within-person variance that defines microbial ecology. Even when statistically significant differences between groups are reported, they may arise from aggregation artifacts rather than true biological divergence [11]. Interpretation is further complicated by compositional data properties: because 16S rRNA profiles reflect relative, not absolute, abundances, apparent differences may emerge simply from changes in one taxon’s proportion rather than in the absolute abundance of others [12,13]. The use of single samples per individual amplifies these issues, preventing assessment of whether observed “differences” are stable, cyclical, or stochastic fluctuations within an individual’s normal range [14,15]. Without longitudinal validation, inferences drawn from group means risk overstating the diagnostic or etiologic relevance of specific taxa or urotypes [16–18].

Interpretive errors also arise when statistical associations are treated as evidence of causal or diagnostic relationships. For example, defining “dysbiosis” as deviation from the composition most frequently observed among asymptomatic participants presumes that relative frequency equates to biological optimality, yet longitudinal evidence from other body sites demonstrates that “healthy” microbial configurations are often individually unique and dynamically maintained [14,19]. In the urinary tract, limited longitudinal data suggest similar individuality and instability [20].

Labeling urotypes as “healthy” or “pathogenic” on the basis of cross-sectional prevalence imposes a static, taxonomic interpretation on a dynamic ecological system. Ecological theory instead defines system health in terms of stability, resilience, and functional redundancy, rather than taxonomic diversity [21] or predominance [22,23]. As recently as 2022, differences between cross-sectional 16S results from groups of individuals with and without urological diagnoses were incorrectly characterized as “changes” [7]. This exemplifies the error of applying a dynamic label to a static observation. Such interpretive shortcuts propagate an illusion of categorical microbial states that do not reflect individual trajectories over time [24].

To address these conceptual, methodological, and interpretive weaknesses, longitudinal studies are required to quantify normal intra-individual variability and test whether group-level inferences are borne out within individuals. A longitudinal design allows examination of temporal stability, transition probabilities, and the persistence of community structure across repeated samples. In the context of SCI/D, where the underlying neurogenic bladder dysfunction, bladder management method, antibiotic exposure, and host physiology interact to shape microbial dynamics, understanding this variability is essential for distinguishing pathological perturbations from normal fluctuation. Without such evidence, the clinical translation of urobiome findings remains speculative.

The present study addresses two related questions: (1) What is the range of normal within-person variability in 16S rRNA-based urobiome profiles among asymptomatic individuals with NLUTD due to SCI/D? (2) Are group-level characterizations of “healthy” or “pathogenic” urobiome profiles, derived from cross-sectional studies, consistent with within-person patterns observed over time? To answer these questions, we analyzed 16S rRNA profiles from each of 92 individuals with NLUTD due to SCI/D, with samples collected at least two weeks apart, all obtained during asymptomatic periods. This descriptive design enables direct evaluation of genus-level variability and urotype stability within individuals, quantifying the extent to which prior group-based inferences align with longitudinal reality.

## Methods

### Participant recruitment

This is a prospective cohort study of people ages 18 and older with SCI/D (including SCI, multiple sclerosis, and spina bifida) who use intermittent catheterization for bladder management. IRB approval was obtained from our institution (#1124) and written informed consent was obtained from all participants prior to inclusion in the study. We recruited from our system-wide outpatient database and nationally from our SCI research database by email and phone. Enrollment started on 1 June 2020 with the final consent obtained on 14 October 2024.

Our exclusion criteria included: 1) use of oral or intravesical prophylactic antibiotics; 2) psychologic or psychiatric conditions influencing the ability to follow instructions; 3) use of oral or IV antibiotics within the past 2 weeks; 4) sexual activity within the previous 72 hours; 5) menstruating at the time of collection; and 6) participation in another study that could confound results. Use of non-antibiotic urinary prophylaxis (e.g., methenamine) was not an exclusion criterion for participation, as no such agent has demonstrated proven efficacy in this population.

We designed the study to obtain two urine samples, separated by at least two weeks, from eligible individuals who were asymptomatic at the time of sampling. We defined “asymptomatic” as the absence of any symptoms on the Urinary Symptom Questionnaire for Neuropathic Bladder-Intermittent Catheter version (USQNB-IC, described below) at the time of the urine sample and for 72 hours afterward. If participants reported any symptom on the USQNB-IC within 72 hours before the sample, a new sample was scheduled. Participation ended when two samples were obtained with no symptoms developing within 72 hours (or the study ended). The 72-hour post-collection symptom window was a deliberate feature intended to distinguish samples collected in a truly stable asymptomatic state from those collected in a latent or transitional state.

### Symptom assessment

We used the Urinary Symptom Questionnaire for Neuropathic Bladder-Intermittent Catheter version (USQNB-IC [25]), a validated tool designed for use by patients, researchers, and clinicians, to assess symptoms in this population. The USQNB-IC retains “neuropathic bladder,” the terminology current when the instrument was developed and validated [25]; we use “neurogenic lower urinary tract dysfunction (NLUTD)” throughout, consistent with standardized terminology. Participants completed the USQNB-IC at least 4 times per sample: at the time of each urine collection (reporting on symptoms over the prior 3 days), and once on each of the three days after each urine collection to ensure that urine samples were “truly asymptomatic” and not “asymptomatic but about to become symptomatic.” The USQNB-IC was not administered prior to the urine sample, but participants had access to it, and if they reported any USQNB-IC symptom within the 3 days prior to the sample collection, it resulted in rescheduling the sample collection for a later time. The USQNB-IC was administered immediately after each sample (“hour 0”), and at 24, 48, and 72 hours afterwards. If the participant endorsed any of the USQNB-IC symptoms during the 72 hours after the urine acquisition, the urine already collected was considered “pre-symptomatic,” and a repeat urine sampling was scheduled at least 2 weeks later following the same method. Three repeat samples were collected at 7–8 days after the initial sample. No questionnaire data were missing.

### Sample collection

We collected urine samples once the following criteria were met 1) no USQNB-IC symptoms for 72 hours prior to urine collection; 2) no active menstruation; and 3) no sexual activity for 72 hours prior to urine collection. A 50–100 ml urine sample from a new, unused intermittent catheter was obtained under sterile conditions. After collection, urine samples were stored at 4°C until processing. The urine was then aliquoted for use in multiple assays. One aliquot was sent to the local clinical microbiology laboratory within 8 hours of collection, during which time it was kept at 4°C. Urinalysis (UA), urine microscopy, and standard urine culture (SUC) were completed using standard laboratory techniques.

A second aliquot was sent within 6 hours to a local laboratory for preparation for 16S rRNA gene sequencing. At the lab, urine samples were centrifuged at 4°C, 5,000 x g for 20 minutes. The supernatant was aliquoted in 2 ml cryotubes and frozen at −20°C. 10 ml phosphate-buffered saline (PBS) was added to the pellet with the remaining supernatant and then centrifuged (5,000 x g) at 4°C for 20 minutes. The pellets and aspirated PBS wash solution were stored at −20°C. Pellets were later transferred to a partner lab for DNA isolation and 16S rRNA gene sequencing.

### Sample processing

*Urinalysis (UA):* Urine samples were assessed by standard clinical microbiology semiquantitative chemical testing using commercial disposable test strips for glucose, bilirubin, ketone, specific gravity, blood pH, protein, urobilinogen, nitrite, and leukocyte esterase.

*Microscopy:* After centrifuging for 5 minutes, microscopic examination for and quantification of white blood cells, red blood cells, epithelial cells, yeast, bacteria, *Trichomonas vaginalis*, sperm cells, mucous filaments and crystals was performed using standard microbial techniques. UA summaries included nitrites (positive/negative), level of leukocyte esterase, and white blood cell counts (UA results are described fully elsewhere [26]).

*Standard Urine Culture (SUC)*: For SUC, a calibrated loop designed to deliver a known volume (1 mL) of urine was used. First, the sample was mixed thoroughly and the top of the container removed. The calibrated wire-inoculating loop was flamed and allowed to cool. The loop was inserted into the urine sample vertically and the loopful of urine was inoculated onto a MacConkey agar plate. A second loopful was collected and inoculated onto a blood agar plate. The plates were incubated aerobically at 35-37°C for at least 24 hours. The colonies of suspected pathogens were counted, and CFU/mL determined. Species identification was performed using Matrix-Assisted Laser Desorption/Ionization – Time-Of-Flight Mass Spectrometry (MALDI-TOF MS).

*Genome extraction:* Depending on the size of the pelleted material, genomic DNA was isolated either with the DNeasy Kit (Qiagen) using manufacturer’s protocol for Gram-negative bacteria or with the QIAmp DNA Micro Kit (Qiagen) using the manufacturer’s protocol for DNA isolation from urine. Purified DNA was quantified using NanoDrop spectrophotometer (Thermo Fisher Scientific). Fractions of human and bacterial DNA in each sample were determined using Femto Human and Femto Bacterial DNA quantification kits (Zymo Research), according to manufacturer’s instructions.

*16S rRNA gene sequencing:* The V4 region of the 16S rRNA gene was amplified using primers 5’-TCGTCGGCAGCGTCAGATGTGTATAAGAGACAGGTGCCAGCMGCCGCGGTAA-3’ and 5’-GTCTCGTGGGCTCGGAGATGTGTATAAGAGACAGGACTACHVGGGTWTCTAAT-3’ and the following reagent concentrations: 20 mM Tris-HCl (pH 8.4), 50 mM KCl, 1.5 mM MgCl_2_, 200 µM of each dNTP, 2 µM of each primer, 1% glycerol, 0.3 U AccuPrime Taq polymerase (Thermo Fisher Scientific) and 25 ng of template DNA in 20 µl total volume. Amplification conditions were 2 minutes at 95°C initial denaturation followed by 30 cycles of 20 seconds denaturation at 95°C, 15 seconds annealing at 55°C and 5 min. extension at 72°C, and a 5-minute final extension at 72°C. Amplification products were purified with the AMPure XP system (Beckman Coulter), and their size was verified with the DNA 1000 Kit (Agilent). Indexing and pooling of amplification products was carried out according to Illumina’s 16S Metagenomic Sequencing Library Preparation protocol. The resulting library was sequenced using Illumina MySeq Reagent Kit v2 (500 cycles).

### Analysis

*Taxonomic identification:* High-quality reads were obtained by trimming low-quality sequences using Cutadapt [27]. The DADA2 software [28] was then used to process the reads through filtering, dereplicating, removing chimeras, and constructing an amplicon sequence variant (ASV) abundance table. ASVs were annotated using the Bayesian LCA (BLCA) method [29] and the NCBI database. ASVs with BLCA confidence scores below 70% were labeled “unknown.” Additionally, ASVs with total counts below the sample number threshold were excluded. Contaminant ASVs were identified using control samples and were determined via a scoring method that encompasses results from Decontam [30], nonparametric statistical testing (Kruskal-Wallis p < 0.5), mean comparison (i.e., mean counts of samples were less than mean counts of extraction controls), and whether sample read counts were less than the 5x greater than extraction control counts. Samples yielding fewer than 1,000 genus-assigned reads were excluded. Decontaminated ASV counts were used for downstream analysis.

*Bioinformatic Analysis:* 16S results were summarized quantitatively and qualitatively (see next section). Within individuals for each time point, results were summarized with a set of alpha (within-samples) diversity indices: Observed, Evenness, Chao1, ACE, Shannon and Simpson. Observed is literally the number of ASVs uniquely identified. This index does not address the abundance of any ASV. Chao1 is an index of how many ASVs were uniquely identified, weighted by abundance to ensure that taxa present at lower abundance levels are adequately represented. ACE (Abundance-based Coverage Estimator) also reflects an abundance-based estimate of richness, but ACE includes terms to separate bacteria with greater and lesser levels of abundance in its equation, as well as a ‘coverage estimator’ to more explicitly incorporate the relative abundances of even the least abundant members of the ecosystem. Larger values of these three indices mean a ‘richer’ ecosystem. Evenness values, based on the Pielou equation, describe the uniformity of ASV distribution; values range from 0 to 1 and larger values indicate a more even distribution. The Shannon and Simpson indices measure both richness and evenness together; values for both range from 0–1. Shannon weights the richness by ASV evenness and larger values indicate greater diversity. Simpson emphasizes ASV evenness to a greater extent with larger Simpson values indicating less diversity [31,32], the opposite of the Shannon index. These calculations were performed in R (R 4.3.1). Because 16S rRNA gene sequencing yields compositional data — relative abundances that reflect proportions within a sample rather than absolute microbial quantities — numerical values cannot be interpreted in isolation or compared directly across samples as continuous measurements. The only defensible characterization of urobiome composition from these data is therefore qualitative: whether or not a sample fits a urotype, defined by the predominant genus (i.e., ≥ 50% of total reads [8,18]), and if so, which genus predominates. A sample is classified as no-predominant when no single genus constitutes ≥50% of total reads. *Escherichia* and *Shigella* are reported as a combined genus because the V4 region of the 16S rRNA gene used for sequencing does not provide sufficient resolution to distinguish these two genera.

Across-sample (within-individual) diversity was summarized with the Bray-Curtis dissimilarity index. Ranging from 0 (the pair of samples share all ASV) to 1 (the pair of samples share no ASVs), lower within-individual values suggest compositional consistency over time. Bray-Curtis values were computed in R (R 4.3.1) for each individual’s samples at times 1, 2, and if available, 3. We did not compute them for the 4th sample since only six individuals had 16S rRNA gene sequencing results for this sample.

*Urine Sample Characterization:* To define normal “variability” within person, we characterized urine samples in terms of their symptoms over 72 hours, inflammatory markers, and cultivable bacteria (*i.e.*, based on Table 1, Tractenberg et al. in review 2026 [26]). This manuscript focuses on 16S rRNA gene sequencing results for urine samples that were characterized according to UA and SUC results. A second paper (Tractenberg et al. in review) [26] reports the UA and SUC results for these same samples. Table 1 (reprinted with permission) shows the five groups, which are based on combinations of symptoms, UA, and SUC results. Four of the five groups describe samples where the individual remained asymptomatic for 72 hours after the sample; the fifth group is the only situation where the participant developed at least one symptom within 72 hours of the sample.

**Table 1:** Classification of Urine Samples Based on (lack of) Symptoms, Urinalysis, and Culture in Individuals with NLUTD due to SCI/D (reprinted from Tractenberg [26] et al., in review with permission).

Group	Label	Definition	Notes
**Group 1**	**Standards-Negative**	Individual is asymptomatic for 72h, UA reveals no pyuria (WBC 0–5), LE negative or trace, nitrite negative, and SUC grows anything, but *below* 10^5^ CFU.	Most stringent and conservative “negative”; samples meeting this group will be classified as “negative” for UTI under available criteria.
**Group 2**	**UA-Negative Asymptomatic** ^†^	Individual is asymptomatic for 72h, UA shows no pyuria (WBC 0–5), LE negative or trace, and nitrite negative. SUC not considered.	Aligns with clinical thresholds for urine lacking indicators of “inflammation”.
**Group 3** ^‡^	**Stable Asymptomatic** ^†^	Two consecutive samples from the same individual who remains asymptomatic for 72 hours, at least 2 weeks apart. UA/UC not considered.	An individual who is stably asymptomatic.
**Group 4** ^‡^	**Single Asymptomatic** ^†^	Individual is asymptomatic for 72h after single sample. UA/UC not considered.	Threshold for “asymptomatic” designation.
**Group 5** ^‡^	**Pre-symptomatic** ^†^ *	Individual is asymptomatic at time of sample but develops symptoms within 72h. UA/UC not considered.	Might reflect latent or subclinical activity potentially leading to UTI§; could be early indicator of microbial or inflammatory shift.

Notes.

UA = urinalysis; SUC = standard urine culture. UTI = urinary tract infection. WBC = white blood cell count. LE = leukocyte esterase. CFU = colony forming units. 72h = for 72 hours after sample.

†“Urinary symptoms” defined as any of the 29 symptoms on the Urinary Symptom Questionnaire for Neuropathic Bladder- Intermittent Catheter user (USQNB-IC [25]), 2018 version.

‡Depends on symptoms only. Given the equivocality about the use/utility of urinalysis and standard urine culture for the diagnosis of a UTI, dependence of characterization on results from urinalysis and standard urine culture are not necessarily realistic [33].

* At least one USQNB symptom was endorsed; not an indicator of a UTI diagnosis. No participant gave a urine sample if they reported any symptoms during the 72 hours before the sample was scheduled to happen; sample was rescheduled in this case. Symptom report during the 72 hours before sample reflects the collection protocol rather than USQNB-verified state. Symptom status at collection and for 72 hours afterwards was recorded on the USQNB-IC completed after each sample.

§Diagnostic criteria for UTI require the presence of at least one symptom – otherwise, the case could only be asymptomatic bacteriuria.

Only group 5 (pre-symptomatic) was mutually exclusive with all of the other groups. Each single-asymptomatic (group 4) sample could be one of consecutive asymptomatic samples; but, even if it was not part of consecutive asymptomatic samples (group 3), single-asymptomatic samples could be UA negative (group 2). Only samples that are UA negative can be standards-negative (group 1).

Data collection was performed in REDCap [34]. Data analyses focused on descriptive statistics only, utilizing SPSS v. 29.0 (2024, IBM).16S summaries were calculated using R (R 4.3.1). Raw sequence data is accessible at NCBI BioProject: ID# PRJNA1499220, entitled “Urobiome composition in individuals with spinal cord injury and disease using intermittent catheter”. All other data underlying the results reported here are accessible in the Zenodo repository at https://doi.org/10.5281/zenodo.21878722.

## Results

Each participant contributed between one and four urine samples, collected at least two weeks apart with six exceptions: two repeat collections following laboratory error (5 and 7 days), three repeat collections following a pre-symptomatic sample (7, 7 and 8 days), and one participant whose two samples were 12 days apart. Analyses focused on qualitative summaries of urotypes and quantitative summaries of alpha diversity indices to evaluate within- and between-person variability in the asymptomatic state.

The majority of individuals’ data appear at timepoints 01 and 02 because most of our participants had asymptomatic first and second samples. Some individuals developed symptoms within 72 hours of their first or second sample and so needed to provide a third and/or fourth sample. 4 participants have a single sample in the analysis set — one gave only one sample before the study ended, and for three others the second sample fell below the read-depth threshold — 63 contributed 2, 19 contributed 3, and 6 contributed 4 (total 211). Of the 92 participants whose samples were analyzed, 61 were male and 31 were female. All participants had SCI/D (traumatic SCI, multiple sclerosis, and spina bifida). The participants whose data are summarized in Table 2 have been fully described elsewhere (Tractenberg et al. in review [26]).

**Table 2:** Demographic information for all participants.

Demographics	Males (n = 61) Mean (SD) or count	Females (n = 31) Mean (SD) or count
age	45.51 (15.17) range 18–75	50.03 (14.60) range 23–84
Yrs since dx	15.77 (14.38) range 1–62	21.87 (14.03) range 1–53
etiology	SCI: n = 59 MS: n = 1 SB: n = 1	SCI: n = 22 MS: n = 6 SB: n = 3
Level of Injury§	Cervical: n = 20	Cervical: n = 5
	Thoracic: n = 34	Thoracic: n = 16
	Lumbar n = 6	Lumbar n = 3
	Unknown n = 1	Unknown n = 7

Notes. dx, diagnosis; SCI, spinal cord injury; MS, multiple sclerosis; SB, spina bifida.

§ where injury spans levels, highest level is reported

Fig 1 presents 16S rRNA gene sequencing results in the form of a series of stacked bar charts, one set for each participant. Samples are labeled according to which of the criteria outlined in Table 1 are met. This figure presents the distribution of urotypes, defined by the predominant genus (*i.e.,* ≥ 50% of total reads [8,18]), across both participants and time points.

**Fig 1:**
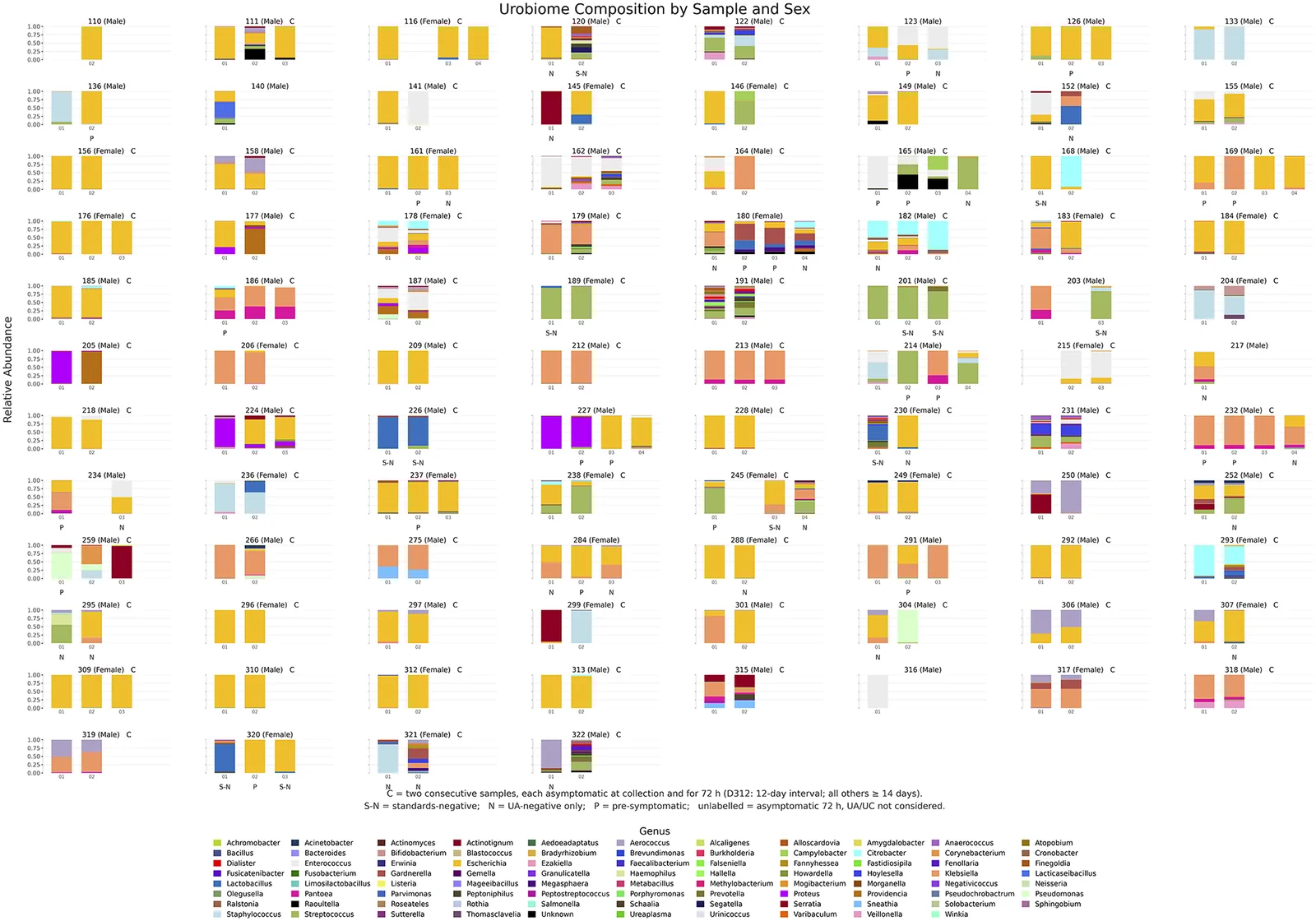
Stacked bar charts showing the 87 most relatively abundant genera detected across the 211 samples yielding 16S rRNA results contributed by the 92 participants. SID = Subject identification number. The y-axis is relative abundance, 0-100%. The category “Unknown” represents all other sequences. At the top of each set of histograms is the participant number and sex. Some are labeled **C** if at least two consecutive samples were obtained when the participant had been asymptomatic for 72 hours afterwards (group 3, Table 1). At the bottom of each bar chart, each sample is labeled using the following scheme: **P** (pre-symptomatic) if the individual was symptomatic within 72 hours afterwards; **N** (negative, UA) if the sample was UA-negative; and **S-N** (SUC and UA negative) if the sample was both SUC-negative and UA-negative. Samples without any designation were asymptomatic for 72 hours (single occurrence), and UA and/or SUC results may not have been available in order to generate the **S or N** identification. Some individuals had to provide an extra sample (due to laboratory error) and were asymptomatic for 72 hours on three (rather than our required two) samples. One participant gave only one sample before the study ended. “Urotype” is defined by the genus observed at ≥50% (i.e., predominant).

The *Gardnerella* urotype was not detected; *Lactobacillus* predominance occurred in four samples from three participants and *Corynebacterium* predominance in one. *Aerococcus*- and *Actinotignum*-predominant samples were also observed. The most prevalent urotypes were predominated by genera considered to possess uropathogenic potential, even when participants remained asymptomatic for at least 72 hours before and after sampling. The most common of these urotypes was the genus *Escherichia/Shigella* (yellow), followed by the genera *Klebsiella* (peach)*, Streptococcus* (green), *Enterococcus* (light gray), and *Staphylococcus* (light blue). “No-predominant” urotypes, within the same individual, often followed or preceded a urotype predominated by a genus with pathogenic potential. Fig 1 is designed to be read holistically: no summary statistic adequately captures the within- and between-person variability. The figure reveals that even qualitative characterizations of urobiome state — the presence or absence of a predominant (≥50%) genus, and its identity — are unstable within individuals over time, and that no pattern observed in one participant reliably predicts the pattern observed in another. Furthermore, the no-predominant category itself is ecologically heterogeneous: some samples classified as no-predominant reflect a truly diverse community with many genera each at low abundance, others reflect two or three genera sharing near-equal dominance, and others reflect a single genus near 49% — biologically near-identical to a predominant sample but classified differently by the ≥ 50% threshold. These distinctions are visible in the figure but lost in any categorical summary.

Some participants demonstrated patterns that illustrate the complexity of within-person urobiome dynamics. For example, one male participant (subject identifier (SID) 111) showed *Escherichia/Shigella* predominance at timepoints 1 (T1) and 3 (T3), with a no-predominant profile at timepoint 2 (T2); another (SID 291) showed *Klebsiella* predominance at timepoints 1 and 3, with *Escherichia/Shigella* predominance at timepoint 2. Whether the flanking or the middle state represents the preferred ecological configuration cannot be determined from these data — the same pattern could reflect either resilience of one state or recurrent displacement by a resource-competitive genus.

To our knowledge, no prior study has empirically tested whether urotype classifications are stable within individuals over time. To examine this, we characterized each participant’s qualitative urotype at each timepoint as predominant (a single genus comprising ≥50% of reads) or no-predominant. Because sampling intervals varied (minimum two weeks, often longer due to scheduling or symptom-related delays), and because the compositional nature of 16S data precludes meaningful quantitative comparison across samples, we describe concordance and discordance in qualitative urotype observations rather than as transitions between biological states. Table 3 summarizes urotype concordance/discordance. Variable discordance was separated into two types, where there was a urotype and it varied (discordant-predominant, DP) or where there was a urotype but not at all timepoints (mixed, M).

**Table 3:** Urotype concordance and discordance across consecutive observations.

Urotype concordance category	T1–T2 (n = 88)	T1–T2–T3 (n = 25)	T1–T2–T3–T4 (n = 6)*
Concordant-predominant (CP)	43 (49%)	9 (36%)	1
Concordant no-predominant (CN)	8 (9%)	1 (4%)	1
Variable (V) — total	37 (42%)	15 (60%)	4
*Discordant-predominant (DP)*	25 (28%)	7 (28%)	2
*Mixed (M)*	12 (14%)	8 (32%)	2

Notes: Urotype is defined as the predominant (≥50%) genus, if there is one. T# = time # (1-4). CP = concordant-predominant (same predominant genus at all observed timepoints in that window). CN = concordant no-predominant (no predominant genus at any observed timepoint). V = variable (any discordance in genus identity or predominance status). DP = discordant-predominant (different genera predominant at different timepoints; subcategory of V). M = mixed (predominant at one timepoint, no-predominant at the other; subcategory of V).

* Because sample is so small, percentages not reported.

The proportion of participants showing variable urotype classifications increased with each additional observation, from 42% at two timepoints to 60% at three and 67% (4/6) at four. Among the 34 consecutive sample pairs that included at least one pre-symptomatic observation, 50% showed discordance in qualitative urotype characterization, compared with 36% among pairs with no pre-symptomatic sample and 40% across all sample pairs.

Quantitative analyses of alpha (within-sample) diversity provided further evidence that the urobiome in this population exhibits substantial normal variability (Fig 2). Diversity metrics included the observed number of taxa, Chao1, ACE, Evenness, Shannon, and Simpson indices. Observed, Chao1, and ACE are measures of richness, with observed being the number of detected ASVs, while both Chao1 and ACE estimate abundance-based richness with differing sensitivities to low-abundance taxa; larger values indicate greater richness. Evenness measures the distribution of ASVs; larger values indicate a more balanced community. Shannon and Simpson integrate richness and evenness, with higher Shannon and lower Simpson values denoting greater diversity.

**Fig 2:**
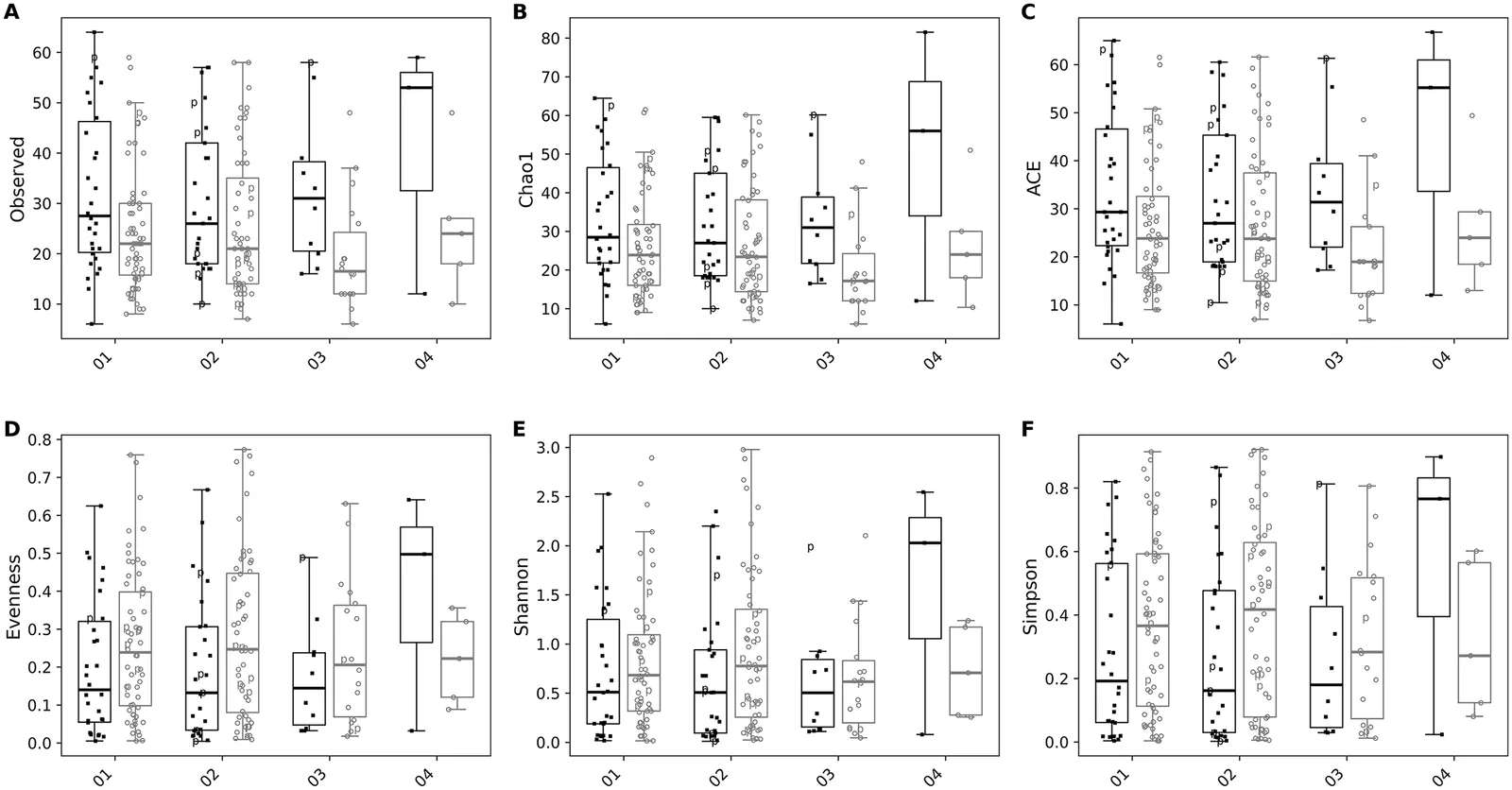
Alpha diversity indices for all samples over time. For all participants, samples are plotted in temporal order, and those collected within 72 hours of symptom onset were marked with a “p.” Samples where an individual was asymptomatic for 72 h after sample are not marked. None of the 4th set of samples met this criterion. Where a dot or a “p” appears in black = Samples from female participants (first plot in every panel at every timepoint), where a dot or a “p” appears in grey = Samples from male participants (second plot in every panel at every timepoint). Y axes reflect different scales (specific to each metric). Metrics: Observed (richness)= the number of detected amplicon sequence variants (ASV); Chao1 and ACE (richness) estimates of abundance-based richness with differing sensitivities to low-abundance taxa; larger values indicate greater richness. Evenness = the distribution of ASVs; larger values indicate more balance. Shannon and Simpson (diversity) combine richness and evenness: higher Shannon and lower Simpson values reflect greater diversity.

Across 92 participants, alpha diversity values spanned nearly the full possible range for each index, regardless of sex or subsequent symptom development. Adult males exhibited lower richness (Observed, Chao1, ACE) than females, but higher Shannon, Simpson and Evenness values, with substantial overlap in every distribution. Diversity distributions differed by index and by sex among asymptomatic participants. In men, Simpson values spanned nearly the full range (0.003–0.921), Evenness ranged from 0.006 to 0.773, and Shannon indices ranged from 0 to 3. In women, Evenness, Simpson, and Shannon indices showed a slightly narrower—but still broad—distribution. In contrast, richness-based measures (Observed number of taxa, Chao1, and ACE) were higher and more variable in females than in males. No consistent pattern was observed between alpha diversity values and asymptomatic versus pre-symptomatic samples, with both high and low diversity values present in each group.

To assess within-person changes in microbial community composition over time, Bray–Curtis dissimilarity indices were computed for each available pair of longitudinal samples (1 vs 2, 2 vs 3, 1 vs 3) separately for males and females (Fig 3). Values of 1 represent completely distinct community membership, whereas 0 indicates identical taxa across the paired samples. Female participants tended to exhibit lower distances when both samples were asymptomatic; only one female pair had both samples pre-symptomatic, and its dissimilarity was high (0.79). Males showed wide variability and bimodal distributions independent of symptom status. Across all comparisons, dissimilarity values ranged from 0 to 1, demonstrating substantial within-person variability in microbial composition over time.

**Fig 3:**
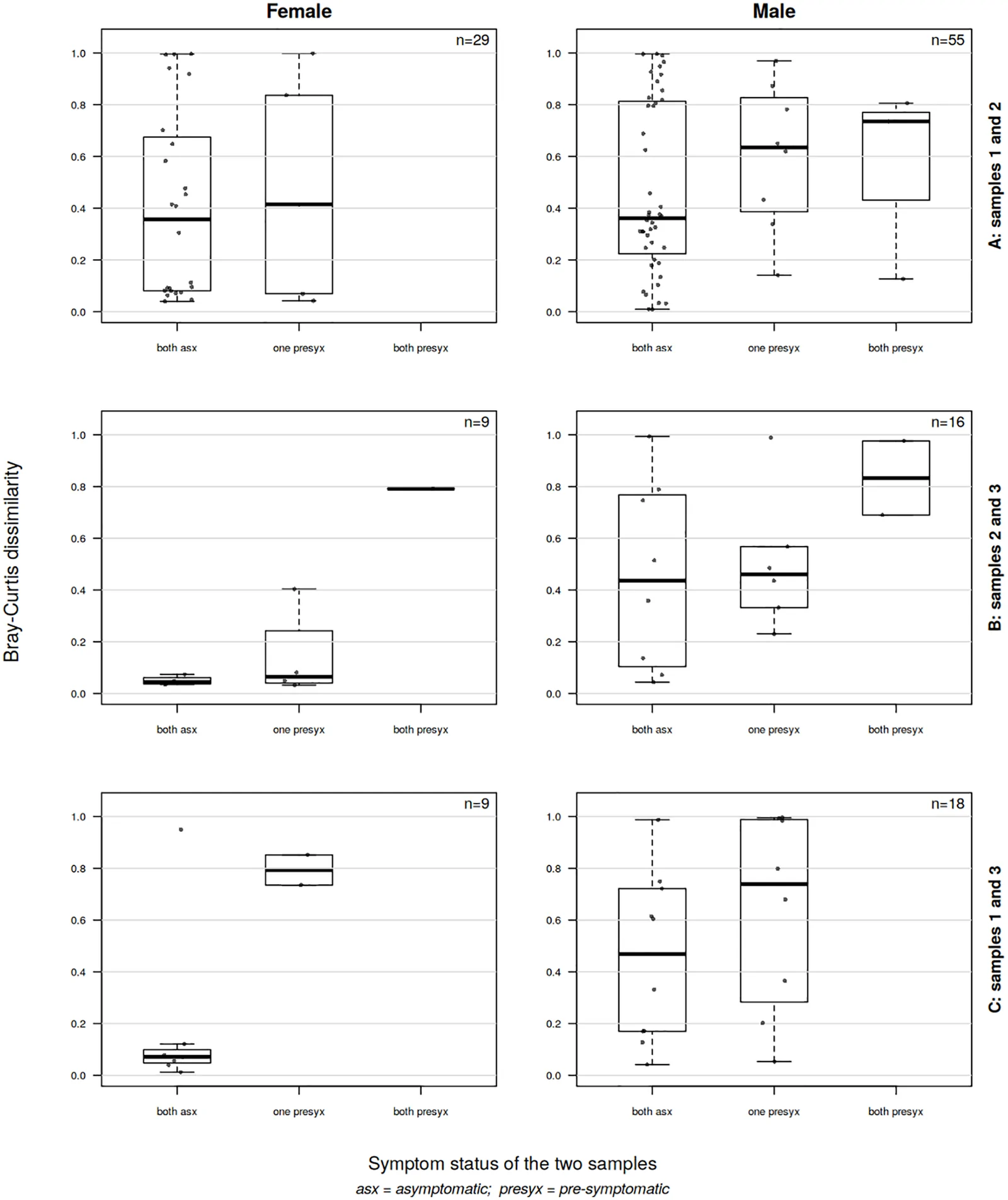
Bray–Curtis dissimilarity between paired urobiome samples from male and female participants. Symptom status: asx = asymptomatic, presyx = pre-symptomatic, asymptomatic at sample but developed at least one USQNB-IC symptom within 72 hours after the sample. Panels A–C show Bray–Curtis distances between pairs of samples (A: 1 vs 2; B: 2 vs 3; C: 1 vs 3). Values near 1 indicate completely different taxa across the two samples, whereas 0 indicates identical community membership. Boxes represent the interquartile range with median lines; individual points show participant-level values. “asx” = asymptomatic for 72 hours before and after the sample; Medians and inter-quartile ranges (IQR) by sex and sample pair appear in S1 Table.

Median Bray-Curtis dissimilarity for the 1 vs 2 comparison was.41 (inter-quartile range (IQR).62) in females and.38 (IQR.56) in males; values for comparisons involving a third sample are summarized in S1 Table, though small cell sizes (n = 9 females, n = 16–18 males) limit interpretation of those estimates. Among female participants, Bray–Curtis values tended to be lowest when both samples were collected during asymptomatic periods, with intermediate values when one of the paired samples preceded symptoms (n = 5 pairs). In males, Bray–Curtis values for the first two samples were distributed bimodally—some near 0.3 and others above 0.6—regardless of symptom status, suggesting two broad patterns of temporal change rather than a monotonic relationship with symptoms. Comparisons that included a third sample (2 vs 3 or 1 vs 3) revealed extensive variability for both sexes, with values spanning most of the 0–1 range even when both samples were asymptomatic. These findings indicate that within-person microbial community composition fluctuates substantially over time in adults with NLUTD due to SCI/D who use intermittent catheterization, and that the magnitude of this variation is not consistently associated with sex or symptom onset within 72 hours.

## Discussion

This study characterized the urobiome among individuals with neurogenic lower urinary tract dysfunction (NLUTD) due to SCI/D who manage their bladders with intermittent catheterization and who were asymptomatic at the time of sampling, across repeated measures. By analyzing repeated 16S rRNA gene sequence profiles collected during asymptomatic periods, we evaluated whether urobiome features that have been characterized as “healthy” or “potentially pathogenic” in prior cross-sectional studies could be replicated within individuals over time. Our results show that they could not. Assumptions derived from one-time group comparisons—such as predominance of certain genera indicates health or disease, or that diversity indices predict urinary health—were not supported. Instead, we observed wide intra- and inter-individual variability, substantial overlap between asymptomatic and pre-symptomatic states, and a consistent predominance of genera conventionally labeled as “uropathogens” even in the absence of symptoms [35]. It is not clear whether the observed variability is due to the presence of NLUTD in our participants, to time between observations, or a combination of both. These findings collectively challenge long-standing conceptual, methodological, and interpretive assumptions about the urobiome in NLUTD, echoing a recent critique that urobiome research has yet to establish definitive causal or diagnostic frameworks [36].

### Predominance of potential uropathogens in asymptomatic urobiomes

Among these participants with NLUTD using intermittent catheterization, asymptomatic urobiomes were commonly predominated by genera often described as uropathogenic, including *Escherichia/Shigella*, *Klebsiella*, *Enterococcus* and *Staphylococcus.* These findings contrast with prior studies that reported “healthy” urobiomes are often predominated by *Lactobacillus* in adult females [18] and by *Corynebacterium* in males without NLUTD [37]. In our cohort, *Lactobacillus* predominance was observed in only one sample from a female participant and in three samples from two males, and *Corynebacterium* predominance was observed in one male sample. Instead, urotypes predominated by taxa typically considered to be pathogenic were nearly ubiquitous even when participants remained symptom-free for ≥72 hours before and after sampling, suggesting that colonization by these taxa is a normal, stable feature of the urobiome in this population rather than an indicator of pathology. These observations align with accumulating evidence that bacterial species historically regarded as uropathogenic are common constituents of the urobiome among individuals with NLUTD due to SCI/D and may play context-dependent or even commensal roles [3,38]. They also reinforce the argument that microbial presence and abundance alone are insufficient to define infection or health status [39]. These patterns suggest that community structures can include genera traditionally regarded as pathogenic in asymptomatic individuals, and that apparent shifts between urotypes may occur independently of symptoms. Thus, for people with NLUTD due to SCI/D, the mere detection of potential uropathogens cannot be equated with disease and should not automatically trigger antimicrobial intervention. This is also true for post-menopausal females [40,41]. This result has direct implications for diagnostic stewardship: microbial sequencing results must be interpreted in the context of individual clinical trajectories and symptom patterns rather than in comparison with population-level “healthy” norms derived from non-comparable groups.

### Temporal and inter-individual variability

Extending this observation, longitudinal analyses revealed that microbial community composition fluctuates substantially over time, even within asymptomatic states. Urotypes frequently shifted between collections, including transitions among uropathogen-predominant profiles without accompanying symptom changes. Bray–Curtis dissimilarities between sequential samples spanned nearly the full 0–1 range, underscoring that distinct community configurations can exist within the same person across short intervals. These results directly contradict the implicit assumption in cross-sectional analyses that between-group differences reflect stable within-person characteristics. Instead, our findings support an ecological view of the urobiome as a dynamic, adaptive system exhibiting both transient and persistent elements—a pattern consistent with longitudinal observations from prior work [2,14]. In this framework, “health” reflects resilience and recovery to an individual’s baseline configuration rather than conformity to a single taxonomic state. These findings highlight the importance of establishing empirically derived reference ranges for normal variability before deviations can be meaningfully interpreted as abnormal.

### Sex-specific patterns in urobiome composition and diversity

Sex differences in the urobiome were subtle and metric-dependent. Asymptomatic males exhibited greater variability in community evenness and diversity indices, whereas females demonstrated higher richness and broader distributions of Chao1 and ACE values. These findings are consistent with prior reports that sex-linked anatomic and hormonal factors influence urobiome ecology [42], but they also reveal that such differences are subtle and do not constitute qualitatively distinct urobiome types — no pattern observed in males is absent in females, and vice versa. Neither sex showed consistent associations between diversity metrics and symptom onset, and both displayed broad within-person variability over time. The implication is that male and female urobiomes in NLUTD are organized around comparable ecological principles—dynamic equilibrium and functional redundancy—rather than fixed, sex-specific community structures. Recognizing these subtle but reproducible differences will be critical for developing analytic frameworks that distinguish biological from methodological variation in future studies.

### Methodological and interpretive implications

The failure of cross-sectional predictions of unhealthy states based on urotype or uropathogen predominance to hold under longitudinal examination exposes fundamental methodological weaknesses in the current urobiome literature. Group-level summaries and majority-rule classifications obscure intra-individual variability, while compositional data constraints inflate apparent differences between groups. The use of diversity indices as proxies for health assumes that higher diversity is inherently beneficial, an assumption that lacks empirical support in urobiome research. Indeed, systematic reviews have underscored that methodological heterogeneity and cross-sectional study designs limit interpretability in studies of lower urinary tract dysfunction [43]. Our data demonstrate that every diversity level occurs in asymptomatic samples and that multivariate dimension-reduction techniques (e.g., Principal Component Analysis and Sparse Partial Least Squares Discriminant Analysis) can remove biologically relevant information if applied uncritically. Future work should therefore prioritize designs and analytic methods capable of capturing temporal dynamics—such as repeated measures, network modeling, or hidden Markov models—over cross-sectional contrasts of mean relative abundances.

### Clinical and stewardship relevance

These findings have direct consequences for both diagnostic and antibiotic stewardship. Diagnostic stewardship demands that clinical decisions be guided by data that accurately distinguish pathological change from normal variation. In the urobiome, our results confirm that a predominance of traditionally defined uropathogens does not necessarily signify infection [35]. We also found that high or low diversity values are not diagnostically informative since the full range of values were observed in individuals without symptoms for 72 hours before and after sampling. These results suggest that, without longitudinal baselines, there is a risk of labeling normal variability as disease. These results support guidance from the Infectious Diseases Society of America [44] to not routinely screen populations at high risk for recurrent UTI, such as those with NLUTD due to SCI/D with urine culture, as it is known that potential uropathogens may be present in the urine in the absence of symptoms. Doing so may expose people unnecessarily to antibiotics.

The case against routine urobiome or microbiological testing in asymptomatic individuals with NLUTD operates at two levels. Every participant in this study was asymptomatic at collection (verified) and for 72 hours beforehand (patient-reported) — including those later classified as pre-symptomatic. At the moment of testing, no participant was clinically distinguishable from any other, yet nearly all samples showed predominance by genera conventionally labeled uropathogenic. Thus, at the level of the individual test, a clinician with these results from any of these individuals during an asymptomatic encounter would obtain a result that appears “abnormal” by standard interpretive frameworks, yet treatment would be unwarranted in every case. Even samples collected within 72 hours of urinary symptom onset were urobiomically indistinguishable from fully asymptomatic samples — there was no microbial signal that would have justified a treatment decision.

Similarly, repeated testing requires careful consideration. Urotype classifications were variable in 42% of participants at two timepoints, 60% at three, and 67% at four (Table 3). A clinician who tested an asymptomatic patient twice might obtain different results, with no basis for determining which, if either, is clinically meaningful or in fact the “true baseline”. Apparent consistency is no more reassuring: 49% of participants appeared concordant-predominant at two timepoints, but usually with a conventionally uropathogenic genus — a result that cannot be acted upon without symptoms. Routine testing in asymptomatic individuals with NLUTD will therefore reliably produce either a consistent result that cannot be acted upon, or an inconsistent result that also cannot be acted upon. Symptoms remain the only reliable trigger for clinical action.

This understanding is critical to antibiotic stewardship, of which a key element is avoidance of overtreatment of asymptomatic bacteriuria, particularly in populations such as NLUTD where colonization is common. Recent reviews have emphasized that progress in defining the urobiome’s clinical relevance depends on disentangling colonization from infection [8], which our findings support. By defining the empirical range of normal urobiome variability, this study provides the reference context necessary to interpret urobiome findings responsibly and to reduce inappropriate antimicrobial use.

### Limitations and future directions

Several limitations of our study must be considered. Although the study included 92 participants and multiple time points, the number contributing three (n = 19) or four (n = 6) samples was smaller, which may limit detection of longer-term trends. Sampling occurred at intervals of at least two weeks, with six shorter intervals of 5–12 days arising from repeat collections following laboratory error or a pre-symptomatic sample, leaving short-term fluctuations unobserved. The study was conducted at a single site, and solely from individuals who use intermittent catheterization as the principal bladder management method. We did not collect data on non-antimicrobial prophylaxis, catheterization frequency, hydration status, bowel management, or dietary patterns, each of which may independently contribute to within-person variability in urobiome composition. Similarly, host immune or inflammatory biomarkers were not obtained; and we did not analyze associations with underlying diagnosis or time since onset of NLUTD. We relied on patient report of no symptoms in the 72 hours prior to urine sample, and excluded samples from individuals actively taking antibiotics at the time of sample (n = 3) and samples yielding fewer than 1,000 genus-assigned reads were also excluded (n = 10). The distribution of read depths was strongly bimodal: the ten excluded samples yielded 10–421 reads and the next lowest retained sample yielded 1,922. The depth exclusion was unsurprisingly more prevalent in samples meeting UA negative (N) criteria: 6 of 31 N samples fell below threshold. Future studies should incorporate these variables to better disentangle biological from behavioral sources of temporal variation. Sequencing was limited to 16S rRNA genus-level taxonomic identification; deeper metagenomic or functional analyses could reveal additional insights into metabolic or ecological stability. Only one of our participants developed a UTI within 24 hours of their sample -definition of “UTI” in that case was not available to us- so our identification of “pre-symptomatic” states, while literally meeting our design criteria, might not reflect true precursors to clinically significant symptoms. Despite these constraints, the consistency of findings across multiple indices and subgroups of adults with NLUTD due to SCI/D who use intermittent catheterization supports the conclusion that the observed variability is authentic — reflecting the genuine biological and experiential complexity of participants’ lives rather than technical noise. This variability is itself the central finding: it challenges convenience assumptions underlying current urobiome research, including the clinical meaningfulness of the ≥ 50% (or any such) predominance threshold and the premise that a urotype observed at one timepoint characterizes an individual’s urobiome or the individual’s health or status. Any clinically relevant signals that may exist within this population are embedded in — and potentially obscured by — this authentic variability, and are unlikely to be recovered by cross-sectional designs or analytical frameworks that assume ecological exchangeability or stable urotypes. Future studies with higher-frequency sampling and species- or strain-level resolution will be essential in order to model transitions, resilience, and recovery dynamics more precisely. Next steps may also include functional assessments of these various urobiomes to better determine the functional roles of both potentially uropathogenic and commensal species.

## Conclusions

Within and across individuals, considerable variability was observed in 16S rRNA gene sequencing results in the absence of urinary symptoms. Asymptomatic states commonly feature potential uropathogens, diversity metrics fail to distinguish health from pre-symptomatic states, and intra-individual variability is substantial. These findings underscore the necessity of redefining “normal” in terms of variability and resilience rather than static composition. Establishing this empirical baseline is a prerequisite for reliable diagnostic interpretation and for advancing both diagnostic and antibiotic stewardship in neurogenic bladder management.

## Supporting information

S1 Table Bray–Curtis dissimilarity between paired urobiome samples, by participant sex and sample pair. Median (interquartile range) of Bray–Curtis dissimilarity for each group; n = number of paired comparisons.
(DOCX)
