# Supplementary material for: Variation in urobiome composition over time in asymptomatic individuals with spinal cord injury and disease using intermittent catheterization
Source: PLoS One. 2026 Oct 30;21(10):e0356391. doi: 10.1371/journal.pone.0356391 (PMC13722290; doi:10.1371/journal.pone.0356391)
Supplement: Supplementary file 1 [file pone.0356391.s001.docx]

**Table S1. Bray–Curtis dissimilarity between paired urobiome samples, by participant sex and sample pair.**

| **Sample pair** | **Sex** | **n** | **Median (IQR)** |
| --- | --- | --- | --- |
| 1 vs 2 | Female | 29 | .41 (.62) |
| 1 vs 2 | Male | 55 | .38 (.56) |
| 2 vs 3 | Female | 9 | .05 (.04) |
| 2 vs 3 | Male | 16 | .50 (.45) |
| 1 vs 3 | Female | 9 | .08 (.68) |
| 1 vs 3 | Male | 18 | .61 (.61) |

*Values are the median (interquartile range) of Bray–Curtis dissimilarity for each group; n = number of paired comparisons. IQR = Q3 − Q1, with quartiles estimated by linear interpolation (type 7).* Table created with R (R 4.3.3).
