# Supplementary material for: Variation in urobiome composition over time in asymptomatic individuals with spinal cord injury and disease using intermittent catheterization
Source: PLoS One. 2026 Oct 30;21(10):e0356391. doi: 10.1371/journal.pone.0356391 (PMC13722290; doi:10.1371/journal.pone.0356391)
Supplement: Supplementary file 2 [file pone.0356391.s003.docx]

**Responses to reviewers**

*1. Please ensure that your manuscript meets PLOS ONE's style requirements, including those for file naming. The PLOS ONE style templates can be found at*

*https://journals.plos.org/plosone/s/file?id=wjVg/PLOSOne_formatting_sample_main_body.pdf and https://journals.plos.org/plosone/s/file?id=ba62/PLOSOne_formatting_sample_title_authors_affiliations.pdf*

done.

*2. Please include a complete copy of PLOS’ questionnaire on inclusivity in global research in your revised manuscript. Our policy for research in this area aims to improve transparency in the reporting of research performed outside of researchers’ own country or community. The policy applies to researchers who have travelled to a different country to conduct research, research with Indigenous populations or their lands, and research on cultural artefacts. The questionnaire can also be requested at the journal’s discretion for any other submissions, even if these conditions are not met. Please find more information on the policy and a link to download a blank copy of the questionnaire here: https://journals.plos.org/plosone/s/best-practices-in-research-reporting. Please upload a completed version of your questionnaire as Supporting Information when you resubmit your manuscript.*

done

*3. We noted in your submission details that a portion of your manuscript may have been presented or published elsewhere. This manuscript has not been published previously and is not under consideration elsewhere. It has been submitted as a preprint to medRxiv (MS ID#: MEDRXIV/2026/347815).*

*Please clarify whether this [conference proceeding or publication] was peer-reviewed and formally published. If this work was previously peer-reviewed and published, in the cover letter please provide the reason that this work does not constitute dual publication and should be included in the current manuscript.*

As is recommended in the PLOS One submission system, a preprint of the manuscript under review was submitted to MedRxiv. We were given a DOI, but the paper has not been peer-reviewed, nor has it been formally published beyond getting this DOI.

*4. We note that you have indicated that there are restrictions to data sharing for this study. For studies involving human research participant data or other sensitive data, we encourage authors to share de-identified or anonymized data. However, when data cannot be publicly shared for ethical reasons, we allow authors to make their data sets available upon request. For information on unacceptable data access restrictions, please see http://journals.plos.org/plosone/s/data-availability#loc-unacceptable-data-access-restrictions.*

*Before we proceed with your manuscript, please address the following prompts:*

*a) If there are ethical or legal restrictions on sharing a de-identified data set, please explain them in detail (e.g., data contain potentially identifying or sensitive patient information, data are owned by a third-party organization, etc.) and who has imposed them (e.g., a Research Ethics Committee or Institutional Review Board, etc.). Please also provide contact information for a data access committee, ethics committee, or other institutional body to which data requests may be sent.*

There are no ethical or legal restrictions.

*b) If there are no restrictions, please upload the minimal anonymized data set necessary to replicate your study findings to a stable, public repository and provide us with the relevant URLs, DOIs, or accession numbers. Please see http://www.bmj.com/content/340/bmj.c181.long for guidelines on how to de-identify and prepare clinical data for publication. For a list of recommended repositories, please see https://journals.plos.org/plosone/s/recommended-repositories. You also have the option of uploading the data as Supporting Information files, but we would recommend depositing data directly to a data repository if possible.*

*Please update your Data Availability statement in the submission form accordingly.*

We are in the process of finalizing multiple manuscripts on the same data set/research project. Creating an anonymized final data set is on our to-do list but has a lower priority than getting papers into peer review. Since this journal encourages, and does not require, that the data are available in order to secure peer review, we left data availability in the lower priority task set. If, after the paper is published but before we have a chance to create the final data set for public availability any interested investigator requests it, that would bump up the priority on making the set publicly available. However, in revising the paper in response to reviewers we have generated additional data (on patterns in the urobiome results over time). So in addition to prioritizing getting the manuscript ready, additional data must be added to the final data set - meaning we can't make the data available until the paper is accepted and we know no additional variables will be created.

*5. Please provide a complete Data Availability Statement in the submission form, ensuring you include all necessary access information or a reason for why you are unable to make your data freely accessible. If your research concerns only data provided within your submission, please write "All data are in the manuscript and/or supporting information files" as your Data Availability Statement.*

We haven't had time to accomplish this yet, and have new data to add now, as noted above.

*6. When completing the data availability statement of the submission form, you indicated that you will make your data available on acceptance. We strongly recommend all authors decide on a data sharing plan before acceptance, as the process can be lengthy and hold up publication timelines. Please note that, though access restrictions are acceptable now, your entire data will need to be made freely accessible if your manuscript is accepted for publication. This policy applies to all data except where public deposition would breach compliance with the protocol approved by your research ethics board. If you are unable to adhere to our open data policy, please kindly revise your statement to explain your reasoning and we will seek the editor's input on an exemption. Please be assured that, once you have provided your new statement, the assessment of your exemption will not hold up the peer review process.*

We have a data sharing plan and have not had the time to implement it. If it is the case that this journal requires data to be available if that isn't legally/ethically restricted, then the policy should say "required" instead of "encouraged".

*7. We note that the grant information you provided in the ‘Funding Information’ and ‘Financial Disclosure’ sections do not match.*

*When you resubmit, please ensure that you provide the correct grant numbers for the awards you received for your study in the ‘Funding Information’ section.*

This research was funded by grant is Department of Defense grant # W81XWH-19-1-0541.

*8. Thank you for stating the following financial disclosure:*

*Department of Defense grant # W81XWH-19-1-0541*

*Please state what role the funders took in the study. If the funders had no role, please state: "The funders had no role in study design, data collection and analysis, decision to publish, or preparation of the manuscript."*

*If this statement is not correct you must amend it as needed.*

*Please include this amended Role of Funder statement in your cover letter; we will change the online submission form on your behalf.*

done.

*9. Please amend your authorship list in your manuscript file to include author Rochelle E. Tractenberg, Suzanne L. Groah, Ethan Newcomb, Mark Khemmani, Cara Joyce, Alan J Wolfe, Christopher R Riegner.*

done.

*10. Please upload a new copy of Figure 2, 3 as the detail is not clear. Please follow the link for more information: https://journals.plos.org/plosone/s/figures*

In response to reviewers we have eliminated figure 1 (bar graph with sample type distribution). Now figure 1 is the bar charts and Figures 2A, 2B, 2C are the box plots. All figures have been re-uploaded as .tiff files.

*11. If the reviewer comments include a recommendation to cite specific previously published works, please review and evaluate these publications to determine whether they are relevant and should be cited. There is no requirement to cite these works unless the editor has indicated otherwise.*

NA

*Additional Editor Comments :*

*Please provide a point‑by‑point response to the reviewers' comments.*

done.

Reviewer #1: Key takeaways for the authors

*This manuscript reports a prospective cohort study of adults with NLUTD due to SCI/D using intermittent catheterization, with repeated urine sampling during carefully verified asymptomatic periods and detailed 16S rRNA-based characterization of the urobiome.*

*Key takeaways are:*

*1. Asymptomatic individuals often have urobiomes dominated by genera traditionally labeled as uropathogens, with substantial intra and inter individual variability over time.*

*2. Alpha diversity indices and Bray–Curtis dissimilarity measures do not reliably distinguish asymptomatic from “asymptomatic at collection with subsequent symptoms” samples.*

*3. Cross-sectional group-level inferences about “healthy” or “pathogenic” urotypes are not supported when examined longitudinally within individuals in this NLUTD population.*

*The Introduction is conceptually fine. I have two suggestions. First, you might more explicitly ground the rationale in current practice and the need for evidence about when urobiome testing is truly useful versus when it may be costly with marginal benefit. In particular, early adoption of promising new diagnostic technologies that are not clearly superior to existing guideline-based approaches can lead to misallocation of resources. Second, the specific aims could be stated more clearly. I suggest moving a concise statement of the two research questions earlier in the Introduction and explicitly stating that the planned analyses are descriptive rather than inferential (no hypothesis tests or models). Clarifying this early will help readers interpret later sections.*

Thank you for this thorough and constructive review. We appreciate the reviewer’s careful reading of the manuscript and respond to each point below. Thank you also for this suggestion; we have done this (lines 53-55 set up the research questions).

Major comments

*1. “Presymptomatic” terminology and interpretation*

*Samples are labeled “presymptomatic” when any USQNB-IC symptom appears within 72 hours, but it is not clear these represent imminent UTI rather than non infectious symptom fluctuations. Consider using more neutral terminology (e.g., “asymptomatic at collection with subsequent symptoms”) and consistently framing analyses as comparing asymptomatic vs symptom-onset–proximate samples, not confirmed pre-UTI states. Where possible, briefly indicate whether these episodes led to clinical encounters or antibiotic treatment.*

We respectfully retain the “presymptomatic” label and disagree that a more neutral term is warranted, or even possible given our design. “Presymptomatic” is not an inference about UTI etiology or clinical trajectory—it is an operationally-defined category built into the study design. Participants were classified as presymptomatic when they were symptom-free at the time of urine collection (and for the 72 hours prior) but endorsed at least one USQNB-IC symptom within the 72 hours following collection. The 72-hour post-collection symptom window was a deliberate design feature, intended to distinguish samples collected in a truly stable asymptomatic state from those collected in a latent or transitional state. This classification depends entirely on symptoms, not on clinical encounter, culture result, or antibiotic treatment. No participant received a UTI diagnosis or antibiotic treatment on the basis of USQNB-IC endorsement alone; symptom endorsement triggered rescheduling of the next sample, not a clinical intervention. We have clarified this in the text [lines 156-158], and in the description of Group 5 in Table 1] to make it explicit that “presymptomatic” refers to proximity to symptom onset.

*2. Urotype definition and stability*

*Urotypes are defined as a predominant genus ≥50% of reads, but stability is described mostly qualitatively. It would help to:*

*• Explicitly define “no-predominant” urotypes and how Escherichia/Shigella were combined.*

*• Provide simple descriptive summaries: proportion of participants whose urotype remained the same across two asymptomatic samples; frequency of transitions between major urotypes; how often transitions occurred in pairs that included symptom-onset–proximate samples.*

Thank you—these are useful clarifications. We have addressed each sub-point as follows.

First, we have added an explicit definition of “no-predominant” urotype to the Methods [lines 272-282]: a sample is classified as no-predominant when no single genus constitutes ≥50% of total reads. Second, we have clarified that *Escherichia* and *Shigella* are reported as a combined genus because the V4 region of the 16S rRNA gene used for sequencing does not provide sufficient resolution to distinguish these two genera [280-282].

Third, and most substantively, we have added urotype concordance and discordance summaries to the Results [lines 365-392]. To our knowledge, the present study is the first to empirically test whether urotype classifications are stable within individuals over time. The urotype concept was defined cross-sectionally, without evidence that it captures a stable, characteristic feature of an individual’s urobiome.

Because sampling intervals varied (minimum two weeks, or longer due to scheduling or symptom-related delays), and because the compositional nature of 16S data precludes meaningful quantitative comparison across samples, we describe concordance and discordance in qualitative urotype observations rather than characterizing differences over time as transitions between biological states.

The proportion of participants showing variable urotype classifications increased with each additional observation: from 41% at two timepoints to 61% at three and 75% at four. This pattern directly supports the paper’s central argument: two observations — and certainly one — substantially underestimate within-person urobiome variability. Several participants who appeared concordant-predominant at two observations revealed variation at three, demonstrating that apparent stability based on limited sampling can be misleading. We also note that the no-predominant category is itself ecologically heterogeneous: some samples reflect a truly diverse community with many genera at low abundance, others reflect two or three genera sharing near-equal dominance, and still others reflect a single genus at ~49% — biologically near-identical to a predominant sample but classified differently by the ≥50% threshold. These distinctions are visible in Figure 1 but are lost in any categorical summary, which is one reason the figure cannot be simplified without losing scientifically meaningful information. We have added a new table (Table 3, starting at line 386) that summarizes this variation.

*3. Presentation of diversity and Bray–Curtis results*

*The alpha diversity and Bray–Curtis results would benefit from more concrete summary statistics in text or a concise table (e.g., medians and IQRs by sex and symptom category for each time pair). This would make patterns like lower Bray–Curtis distances between asymptomatic pairs in females and the bimodal pattern in males easier to appreciate, without adding formal hypothesis testing.*

We agree that summary statistics strengthen the presentation. We have added medians and interquartile ranges for each alpha diversity index by sex and timepoint as a supplementary table, with a brief pointer to this table in the Results text ([lines 449-452] and Figure 3 legend [lines 446-447]. We did not stratify by symptom category (asymptomatic vs. presymptomatic), because the number of presymptomatic samples is small and cell-level summaries would be unreliable and potentially misleading. We note this constraint explicitly in the Results. For Bray–Curtis dissimilarity, the same supplementary table includes medians and IQRs by sex and sample pair (1 vs 2, 2 vs 3, 1 vs 3). No inferential statistics have been added.

*4. Relationship to the companion “urinary markers” paper*

*Because Table 1 and Figure 1 are reprinted and central to interpreting sample groups, please ensure this paper is self-contained:*

*• Briefly summarize how Groups 1–5 align with common clinical UTI definitions.*

*• State clearly what is unique to this manuscript (16S-based urobiome variability) versus what is covered in the companion work (urinalysis, culture, biomarkers).*

*You might consider moving Figure 1 to the supplement and retaining a short textual summary of group distributions by sex.*

The two papers are self contained with Table 1 shared between them, and only the whole sample demographics appearing in both papers (the other paper has demographic descriptions for those contributing to every subset). We now differentiate the two papers explicitly [lines 295-298]. We agree Figure 1 is not essential to the urobiome findings presented here, so we have removed it.

We respectfully decline to align Groups 1–5 with “common clinical UTI definitions,” as there is no single accepted standard clinical UTI definition in this population. Mapping our groups onto variable external criteria would introduce exactly the kind of false equivalence the paper seeks to challenge. Notably, UTI definitions vary over time and expert group in terms of UA, UC, and which symptoms to consider. Finally, since every sample is taken when the participant has no symptoms, UTI diagnosis is not a plausible for explicating the groupings.

*Policy and clinical-practice implications*

*The Discussion already connects the findings to diagnostic and antibiotic stewardship, and this could be strengthened with a few explicit statements about implications for policy and practice:*

*• The authors might consider adding that their findings argue against routine culture or advanced microbiome testing in asymptomatic individuals with NLUTD due to SCI/D, since such testing will frequently identify “abnormal” taxa that actually fall within the normal range of intra individual variability and are unlikely to warrant treatment.*

*• It would be helpful if the Discussion explicitly linked these results to diagnostic and antibiotic stewardship policies by emphasizing that symptom-based criteria should remain the primary trigger for UTI treatment decisions, with culture and sequencing data interpreted only in the context of each patient’s longitudinal clinical trajectory rather than population-level “healthy” norms.*

*• The authors might also wish to note that future guideline and research agendas should prioritize longitudinal, patient-level designs that define individualized baselines and actionable thresholds for change, rather than additional cross-sectional sequencing studies that are unlikely to yield clinically usable definitions of “healthy” or “pathogenic” urotypes.*

We agree and have strengthened the Discussion about clinical practice and stewardship along these lines. The argument [580-602] is made at the level of an individual test and when considering repeated testing. We conclude [lines 599-602], "Routine testing in asymptomatic individuals will therefore reliably produce either a consistent result that cannot be acted upon, or an inconsistent result that also cannot be acted upon. Symptoms remain the only reliable trigger for clinical action, and future research agendas should prioritize longitudinal, patient-level designs over additional cross-sectional sequencing studies."

Minor comments

*1. Abstract*

*The abstract is dense and somewhat repetitive. Streamlining repeated phrases and adding one or two specific quantitative descriptors (e.g., proportion of asymptomatic samples with Escherichia/Klebsiella/Enterococcus predominance; typical range of Bray–Curtis values) would make the findings more concrete and concise.*

Thank you for these suggestions, we have revised the abstract [lines 20-26].

*2. Methods*

*• Clarify the recruitment setting and whether participants were consecutively approached or recruited by convenience.*

*• Indicate how missing USQNB-IC questionnaires were handled (e.g., rescheduling vs exclusion).*

*• Specify whether non-antibiotic urinary prophylaxis (such as methenamine) was allowed.*

We have added clarifying text to the Participant Recruitment section of the Methods addressing all three sub-points. Participant recruitment is now described [lines 138-148]. All participants completed the USQNB-IC questionnaire at each attended visit; no questionnaire data were missing [line 171]. Use of non-antibiotic urinary prophylaxis was not an exclusion criterion, as no such agent has demonstrated proven efficacy in this population. [lines 146-148]

3. Figures and tables

• Figure 2 is informative but visually dense. Consider simplifying the main-text version (e.g., combining low-frequency genera into “other”) and providing a full version in the supplement.

• Table 2 could be complemented by a short table summarizing numbers of samples per participant (1, 2, 3, 4) and counts of asymptomatic versus symptom-onset–proximate samples.

Figure 2 is now Figure 1. It is intentionally dense, and we have added narrative [lines 346-392] about the difficulties of summarizing what appears - not because the figure is dense but because there is simply no coherent pattern to be discerned. Figure 1 also presents the number of samples together with both the pattern of urobiome predominance (or not) and the sample types. While we could add a table as you suggest, it would imply that patterns we summarize are exchangeable - that a simple sum of observation counts would be accurately descriptive - which is inconsistent with the manuscript's core message.

*4. Terminology and consistency*

*Ensure consistent use of “NLUTD due to SCI/D” and of labels for “asymptomatic” vs “symptom-onset–proximate” samples in text and figure legends. A brief definition of “urotype” and “predominance category” early in Methods would help readers follow later results.*

Urotype and predominance have now been clarified in response to earlier comments; asymptomatic and presymptomatic are clearly and consistently used throughout. We have reviewed the manuscript for terminological consistency; we have standardized to 'NLUTD due to SCI/D' throughout, using the full term at first reference in each section and 'NLUTD' alone thereafter for readability. Sample labels (asymptomatic, presymptomatic) are applied consistently in text and figure legends. We have also added a definition of “urotype” and “predominance category” to the Methods [lines 272-282]. Note that we retain “presymptomatic” rather than “symptom-onset–proximate” for the reasons set out in our response to Major Comment 1.

*Reviewer #2: The manuscript addresses an important and clinically relevant topic regarding longitudinal variability of the urobiome in individuals with SCI/D using intermittent catheterization. The longitudinal design and repeated sampling strategy are strengths of the study and provide valuable insight into intra-individual microbial variability.*

*The methodology is generally appropriate and clearly described, and the conclusions are mostly supported by the presented findings. The manuscript is well written and logically organized.*

Thank you for the careful and supportive reading of this manuscript. We respond to each suggestion below.

*I have a few minor suggestions for improvement:*

*1. The authors may consider further clarifying the rationale for defining “pre-symptomatic” samples, especially since no participant developed clinically confirmed UTI.*

We have added clarifying text to the Methods and to the description of Group 5 in Table 1. (See also response to Reviewer 1). “Presymptomatic” is an operationally defined category, not an inference about UTI risk or clinical trajectory. It refers specifically to samples collected during a period of verified symptom-freedom that was followed within 72 hours by endorsement of at least one USQNB-IC symptom. The designation is symptom-based only: no participant received a UTI diagnosis or antibiotic treatment on the basis of USQNB-IC symptom endorsement alone, and symptom endorsement triggered rescheduling of the next sample collection rather than a clinical encounter. The label “presymptomatic” therefore means precisely what it says—the sample was collected prior to symptom onset within a specific time frame.

*2. Some figures, particularly the stacked bar plots, are visually dense and may benefit from improved readability or simplified presentation.*

We have removed original Figure 1 (histogram) and now Figure 1 (formerly Figure 2) is intentionally comprehensive. It is designed to be read holistically: the overall impression of variability — the absence of any consistent pattern within or across individuals, the same apparently uropathogenic genera in asymptomatic and presymptomatic samples alike, and the ecological heterogeneity within the no-predominant category — is itself the central evidence of the paper. A simplified version would obscure it. We also note that every time one examines this figure, a different pattern becomes salient — and whenever a sex-specific pattern appears to emerge, an individual of the other sex shows the same pattern. This is a visual demonstration that sex differences in this population do not constitute qualitatively distinct urobiome types, a point that no group-level summary can convey. We have revised the legend for clarity, ensured the figure meets journal technical requirements, and cross-refer readers in the legend to the concordance and discordance summaries added in response to Reviewer 1, Major Comment 2, which provide a quantitative narrative complement.

*3. The discussion could briefly address the potential influence of catheterization frequency, hydration status, or dietary variation on temporal microbiome variability.*

Thank you for this suggestion -we agree. We did not collect data on these individual characteristics. We have added these to the list of limitations [lines 615-619]. Also, we have elaborated on the authenticity of the variability we have documented with respect to patients lived experience - which must be considered in future research rather than ignored, screened out, or 'statistically controlled for' [lines 626-637].

*4. The Data Availability Statement should clarify whether anonymized sequencing data will be publicly accessible immediately upon publication and include repository details if available.*

The research team is in the process of finalizing multiple manuscripts drawing on the same dataset and is working to ensure the data are complete, accurate, and appropriately anonymized prior to deposition. The sequencing data are not under the sole control of the corresponding author; coordination with the sequencing team is required before public deposit can be completed. We anticipate making the data available in a public repository (such as NCBI SRA) upon or shortly after acceptance and will provide the accession number at that time. We will have an updated the Data Availability Statement if this manuscript is accepted (prior to publication).

*Overall, this is a thoughtful and technically sound study that contributes important evidence regarding interpretation of urobiome findings in NLUTD populations.*

We appreciate this attention to our work!
