# Supplementary material for: Variation in urobiome composition over time in asymptomatic individuals with spinal cord injury and disease using intermittent catheterization
Source: PLoS One. 2026 Oct 30;21(10):e0356391. doi: 10.1371/journal.pone.0356391 (PMC13722290; doi:10.1371/journal.pone.0356391)
Supplement: Supplementary file 3 [file pone.0356391.s004.docx]

Responses to Reviewers, R2

We appreciate the reviewer's time and attention to this manuscript. During the interim between their first and second reviews, the laboratory submitted the raw sequences to NCBI so now we can assure them that anonymized sequencing data are publicly accessible. Raw sequence data is accessible at NCBI BioProject: ID# PRJNA1499220, entitled “Urobiome composition in asymptomatic individuals with spinal cord injury and disease using intermittent catheter" (lines 319-321). We are also in the process of creating the data sets and data dictionaries for the data in this paper and the other main paper from these samples (Reference 26, manuscript currently in review at PLOS One).

*Reviewer #1: The authors addressed most major comments thoroughly—urotype concordance, descriptive statistics, and clinical stewardship implications are now substantially strengthened.

Two substantive variances remain:*

*Terminology ("presymptomatic" vs. "asymptomatic at collection with subsequent symptoms"): Clarified but not resolved. The term persists and may still prompt concern about clinical inference.*

We appreciate the reviewer's comment and discussed this terminology at length. We decided that dedicating additional narrative to this descriptor, for so few samples, would detract from the reader's attention while not adding much to the findings because there ended up being so few 'presymptomatic' samples. Moreover, since no one in the study actually developed a UTI within 72 hours, there was no rationale for varying from 'presymptomatic' since it is generically descriptive.

*Groups 1–5 alignment with UTI definitions: Declined.*

We happen to have been working on another manuscript looking at 2024 international consensus UTI reference criteria for UTI diagnosis and applying that algorithm to the same urine samples and individuals as are reported in this manuscript. As is stated in the manuscript, every sample was obtained after 72 hours without symptoms and in the asymptomatic state - and yet the 2024 criteria misclassify the individuals, based solely on the UA and UC results (which are not presented in this paper), as having a possible or probable UTI 60-96% of the time. These findings (currently in final preparation for review elsewhere) underscore the decision not to use UTI definitions since they are - even when current and based on consensus - highly unreliable.

*Reviewer #2: Thank you for the opportunity to review this revised manuscript entitled "Variation in Urobiome Composition Over Time in Asymptomatic Individuals with Spinal Cord Injury and Disease Using Intermittent Catheterization." The study addresses an important clinical problem by investigating longitudinal variability of the urinary microbiome in individuals with neurogenic lower urinary tract dysfunction (NLUTD). The longitudinal design, repeated sampling strategy, and emphasis on distinguishing normal microbial variation from clinically meaningful changes represent notable strengths. The manuscript is well written and provides valuable data that may contribute to improving microbiome-informed diagnosis of urinary tract infection in this population. Nevertheless, several issues should be addressed before the manuscript is suitable for publication.

Major Comments*

*1. Clarify the novelty of the study. Although the manuscript emphasizes the limitations of cross-sectional microbiome studies, the introduction should more explicitly state how this study advances current knowledge beyond previous longitudinal urobiome investigations. A concise statement highlighting the unique contribution and clinical implications would strengthen the rationale.*

Our conclusions (lines 633-640) are explicit without overstating the results or their generalizability (per this reviewer's comments #6 and #8).

*2. Sample size justification. The manuscript includes 94 participants; however, no sample size calculation or justification is provided. Even for a descriptive longitudinal study, the authors should explain how the sample size was determined and discuss its adequacy for addressing the study objectives.*

Since there is no effect size, there is no rationale for a sample size justification. Our objective was to obtain at least two urine samples from 100 asymptomatic individuals meeting inclusion criteria (per our grant proposal) because 100 individuals would more than meet any statistical or inferential assumption about patterns we might observe. Only 94 samples were analyzed via 16S rRNA.

*3. Handling of repeated measurements. While the study is primarily descriptive, participants contributed varying numbers of urine samples over time. The manuscript should explain how unequal numbers of repeated observations were handled and discuss whether this may have influenced the reported variability.*

The study is solely descriptive - not primarily descriptive - and explicitly, no statistical considerations about repeated measurements are included in the manuscript. The study design (lines 330 -335) is explicit about the number of samples - we only asked for two after which an individual remained asymptomatic for 72 hours. It is not feasible to contemplate how this design might have affected variability - and this manuscript is the first to our knowledge ever to even focus on the fact that cross sectional analyses presume that the one-time urobiome result is stable -and to show that this presumption is not supported.

*4. Potential confounding factors. Several factors known to influence the urinary microbiome—including age, sex, underlying neurological diagnosis, duration of disease, catheterization frequency, diet, hydration, bowel management, and prior antibiotic exposure—are not explored. At minimum, the limitations section should acknowledge these potential confounders and discuss their possible impact on microbial variability.*

In lines 602-609 we identify all such factors as unstudied.

*5. Statistical methodology. The exclusive reliance on descriptive statistics limits the strength of the conclusions. Consider including confidence intervals or additional exploratory longitudinal analyses to quantify the stability of microbial composition. Even if inferential analyses are intentionally avoided, the rationale for this decision should be explained more clearly.*

We respectfully disagree that descriptive statistics limit the strength of the findings. No assumptions are made in this manuscript and our argument is that the assumption that a cross sectional urobiome analysis can be leveraged meaningfully - particularly in adults with NLUTD due to SCI/D who use intermittent catheterization - is untenable. With no calculation and solely a plain demonstration of inter and intra personal variability in 16S rRNA urobiome results across 94 individuals, Figure 1 in particular needs no statistical analyses - particularly since our claims are explicitly about there being NO evidence of stability in microbial composition.

*6. Interpretation of the findings. Some conclusions regarding the limitations of cross-sectional studies appear stronger than the data directly support. The discussion should be revised to avoid overgeneralization and acknowledge that the findings are specific to adults with NLUTD using intermittent catheterization.*

We respectfully disagree that our data do not support conclusions about cross sectional designs: cross sectional studies are by their design limited - especially when considering the dynamic nature of the microbiome in any niche. These data support the intuitive/logical conclusion that cross sectional studies are literally limited. Lines 456 and 617 now focus the findings.

*7. Clinical implications. The discussion would benefit from a more detailed explanation of how these findings could influence future diagnostic algorithms for urinary tract infection, antimicrobial stewardship, and personalized microbiome-based clinical decision-making.*

Given reviewer comment 8 below, and the fact that every person in the sample was asymptomatic at the time of urine sampling, the results (wide inter and intra individual variability in summary statistics in the absence of symptoms) are strongly supportive of greater care being used in future development of algorithms and use of the urobiome in clinical decision making, all of which are stated (lines 616-628).

*8. Limitations. The limitations section should be expanded to include the single-center nature of the cohort, limited generalizability to other bladder-management methods, reliance on 16S rRNA sequencing rather than shotgun metagenomics, inability to evaluate microbial function, and the absence of host immune or inflammatory biomarkers.*

Added (lines 602-609)

*Further comments*

*1. The abstract is somewhat lengthy and repetitive. Consider shortening the concluding sentences to improve readability.*

We have edited the abstract for clarity and concision.

*2. Several paragraphs in the Introduction are considerably long and could be divided into shorter sections to improve flow.*

Done (lines 88 and 118)

*3. The Methods section contains duplicated descriptions of DNA extraction/genome extraction. These sections should be consolidated to avoid redundancy.*

Done

*4. Please define all abbreviations at their first appearance and ensure consistent use throughout the manuscript.*

Done

*5. Figure legends should be expanded to allow readers to understand the figures without referring extensively to the main text.*

Done.

*6. Table 2 would be more informative if percentages were reported alongside frequencies where appropriate.*

We respectfully disagree that percentages will be supportive given how few observations fall into most categories in the table; specifically, we have not used percentages where they could manufacture a false sense of precision for the reader. However, we have clarified the column headers.

*7. Please ensure that all cited references are up to date and formatted consistently according to journal style.*

We have corrected incomplete journal names in references 21, 23, and 32, a malformed DOI in reference 16, and standardized DOI formatting and author listing throughout

*8. Carefully proofread the manuscript for minor grammatical inconsistencies and formatting issues, particularly spacing around citations and punctuation.*

We have reviewed and corrected these inconsistencies- thank you for your careful read!

*This manuscript addresses an important and clinically relevant topic and has several methodological strengths, particularly its prospective longitudinal design and rigorous symptom monitoring. The findings provide valuable insight into temporal variability of the urobiome in individuals with SCI/D and have potential implications for future microbiome-informed diagnostics. However, clarification of the methodology, expansion of the discussion, and a more balanced interpretation of the findings are necessary before publication.*

We appreciate this summary and your attention!
