# Supplementary material for: Variation in urobiome composition over time in asymptomatic individuals with spinal cord injury and disease using intermittent catheterization
Source: PLoS One. 2026 Oct 30;21(10):e0356391. doi: 10.1371/journal.pone.0356391 (PMC13722290; doi:10.1371/journal.pone.0356391)
Supplement: Peer review history

# Peer review: Decision Letter 0

**DOI:** 10.1371/journal.pone.0356391.r001

**Authors:** Zongxin Ling

18 May 2026

Dear Dr. Tractenberg,

========================

Please provide a point‑by‑point response to the reviewers' comments.

Please submit your revised manuscript by Jul 02 2026 11:59PM. If you will need more time than this to complete your revisions, please reply to this message or contact the journal office at plosone@plos.org. When you're ready to submit your revision, log on to https://www.editorialmanager.com/pone/ and select the 'Submissions Needing Revision' folder to locate your manuscript file.

- A letter that responds to each point raised by the academic editor and reviewer(s). You should upload this letter as a separate file labeled 'Response to Reviewers'.
- A marked-up copy of your manuscript that highlights changes made to the original version. You should upload this as a separate file labeled 'Revised Manuscript with Track Changes'.
- An unmarked version of your revised paper without tracked changes. You should upload this as a separate file labeled 'Manuscript'.

If you would like to make changes to your financial disclosure, please include your updated statement in your cover letter. Guidelines for resubmitting your figure files are available below the reviewer comments at the end of this letter.

If applicable, we recommend that you deposit your laboratory protocols in protocols.io to enhance the reproducibility of your results. Protocols.io assigns your protocol its own identifier (DOI) so that it can be cited independently in the future. For instructions see: https://journals.plos.org/plosone/s/submission-guidelines#loc-laboratory-protocols. Additionally, PLOS ONE offers an option for publishing peer-reviewed Lab Protocol articles, which describe protocols hosted on protocols.io. Read more information on sharing protocols at https://plos.org/protocols?utm_medium=editorial-email&utm_source=authorletters&utm_campaign=protocols.

As the corresponding author, your ORCID iD is verified in the submission system and will appear in the published article. PLOS supports the use of ORCID, and we encourage all coauthors to register for an ORCID iD and use it as well. Please encourage your coauthors to verify their ORCID iD within the submission system before final acceptance, as unverified ORCID iDs will not appear in the published article. *Only*  the individual author can complete the verification step; PLOS staff *cannot*  verify ORCID iDs on behalf of authors.

We look forward to receiving your revised manuscript.

Kind regards,

Zongxin Ling

Academic Editor

PLOS One

Journal Requirements:

When submitting your revision, we need you to address these additional requirements.

1. Please ensure that your manuscript meets PLOS ONE's style requirements, including those for file naming. The PLOS ONE style templates can be found at

https://journals.plos.org/plosone/s/file?id=wjVg/PLOSOne_formatting_sample_main_body.pdf and https://journals.plos.org/plosone/s/file?id=ba62/PLOSOne_formatting_sample_title_authors_affiliations.pdf

2. Please include a complete copy of PLOS’ questionnaire on inclusivity in global research in your revised manuscript. Our policy for research in this area aims to improve transparency in the reporting of research performed outside of researchers’ own country or community. The policy applies to researchers who have travelled to a different country to conduct research, research with Indigenous populations or their lands, and research on cultural artefacts. The questionnaire can also be requested at the journal’s discretion for any other submissions, even if these conditions are not met.  Please find more information on the policy and a link to download a blank copy of the questionnaire here: https://journals.plos.org/plosone/s/best-practices-in-research-reporting. Please upload a completed version of your questionnaire as Supporting Information when you resubmit your manuscript.

3. We noted in your submission details that a portion of your manuscript may have been presented or published elsewhere. This manuscript has not been published previously and is not under consideration elsewhere. It has been submitted as a preprint to medRxiv (MS ID#: MEDRXIV/2026/347815).

Please clarify whether this [conference proceeding or publication] was peer-reviewed and formally published. If this work was previously peer-reviewed and published, in the cover letter please provide the reason that this work does not constitute dual publication and should be included in the current manuscript.

4. We note that you have indicated that there are restrictions to data sharing for this study. For studies involving human research participant data or other sensitive data, we encourage authors to share de-identified or anonymized data. However, when data cannot be publicly shared for ethical reasons, we allow authors to make their data sets available upon request. For information on unacceptable data access restrictions, please see http://journals.plos.org/plosone/s/data-availability#loc-unacceptable-data-access-restrictions.

Before we proceed with your manuscript, please address the following prompts:

a) If there are ethical or legal restrictions on sharing a de-identified data set, please explain them in detail (e.g., data contain potentially identifying or sensitive patient information, data are owned by a third-party organization, etc.) and who has imposed them (e.g., a Research Ethics Committee or Institutional Review Board, etc.). Please also provide contact information for a data access committee, ethics committee, or other institutional body to which data requests may be sent.

b) If there are no restrictions, please upload the minimal anonymized data set necessary to replicate your study findings to a stable, public repository and provide us with the relevant URLs, DOIs, or accession numbers. Please see http://www.bmj.com/content/340/bmj.c181.long for guidelines on how to de-identify and prepare clinical data for publication. For a list of recommended repositories, please see https://journals.plos.org/plosone/s/recommended-repositories. You also have the option of uploading the data as Supporting Information files, but we would recommend depositing data directly to a data repository if possible.

Please update your Data Availability statement in the submission form accordingly.

5. Please provide a complete Data Availability Statement in the submission form, ensuring you include all necessary access information or a reason for why you are unable to make your data freely accessible. If your research concerns only data provided within your submission, please write "All data are in the manuscript and/or supporting information files" as your Data Availability Statement.

6. When completing the data availability statement of the submission form, you indicated that you will make your data available on acceptance. We strongly recommend all authors decide on a data sharing plan before acceptance, as the process can be lengthy and hold up publication timelines. Please note that, though access restrictions are acceptable now, your entire data will need to be made freely accessible if your manuscript is accepted for publication. This policy applies to all data except where public deposition would breach compliance with the protocol approved by your research ethics board. If you are unable to adhere to our open data policy, please kindly revise your statement to explain your reasoning and we will seek the editor's input on an exemption. Please be assured that, once you have provided your new statement, the assessment of your exemption will not hold up the peer review process.

7. We note that the grant information you provided in the ‘Funding Information’ and ‘Financial Disclosure’ sections do not match.

When you resubmit, please ensure that you provide the correct grant numbers for the awards you received for your study in the ‘Funding Information’ section.

8. Thank you for stating the following financial disclosure:

Department of Defense grant # W81XWH-19-1-0541

Please state what role the funders took in the study. If the funders had no role, please state: "The funders had no role in study design, data collection and analysis, decision to publish, or preparation of the manuscript."

If this statement is not correct you must amend it as needed.

Please include this amended Role of Funder statement in your cover letter; we will change the online submission form on your behalf.

9. Please amend your authorship list in your manuscript file to include author Rochelle E. Tractenberg, Suzanne L. Groah, Ethan Newcomb, Mark Khemmani, Cara Joyce, Alan J Wolfe, Christopher R Riegner.

10. Please upload a new copy of Figure 2, 3 as the detail is not clear. Please follow the link for more information: https://journals.plos.org/plosone/s/figures

11. If the reviewer comments include a recommendation to cite specific previously published works, please review and evaluate these publications to determine whether they are relevant and should be cited. There is no requirement to cite these works unless the editor has indicated otherwise.

Additional Editor Comments :

Please provide a point‑by‑point response to the reviewers' comments.

[Note: HTML markup is below. Please do not edit.]

Reviewers' comments:

Reviewer's Responses to Questions

**Comments to the Author**

1. Is the manuscript technically sound, and do the data support the conclusions?

Reviewer #1: Yes

Reviewer #2: Yes

**********

2. Has the statistical analysis been performed appropriately and rigorously?

Reviewer #1: Yes

Reviewer #2: Yes

**********

3. Have the authors made all data underlying the findings in their manuscript fully available?

The PLOS Data policy

Reviewer #1: Yes

Reviewer #2: Yes

**********

4. Is the manuscript presented in an intelligible fashion and written in standard English?

Reviewer #1: Yes

Reviewer #2: Yes

**********

Reviewer #1: Key takeaways for the authors

This manuscript reports a prospective cohort study of adults with NLUTD due to SCI/D using intermittent catheterization, with repeated urine sampling during carefully verified asymptomatic periods and detailed 16S rRNA-based characterization of the urobiome.

Key takeaways are:

1. Asymptomatic individuals often have urobiomes dominated by genera traditionally labeled as uropathogens, with substantial intra and inter individual variability over time.

2. Alpha diversity indices and Bray–Curtis dissimilarity measures do not reliably distinguish asymptomatic from “asymptomatic at collection with subsequent symptoms” samples.

3. Cross-sectional group-level inferences about “healthy” or “pathogenic” urotypes are not supported when examined longitudinally within individuals in this NLUTD population.

The Introduction is conceptually fine. I have two suggestions. First, you might more explicitly ground the rationale in current practice and the need for evidence about when urobiome testing is truly useful versus when it may be costly with marginal benefit. In particular, early adoption of promising new diagnostic technologies that are not clearly superior to existing guideline-based approaches can lead to misallocation of resources. Second, the specific aims could be stated more clearly. I suggest moving a concise statement of the two research questions earlier in the Introduction and explicitly stating that the planned analyses are descriptive rather than inferential (no hypothesis tests or models). Clarifying this early will help readers interpret later sections.

Major comments

1. “Presymptomatic” terminology and interpretation

Samples are labeled “presymptomatic” when any USQNB-IC symptom appears within 72 hours, but it is not clear these represent imminent UTI rather than non infectious symptom fluctuations. Consider using more neutral terminology (e.g., “asymptomatic at collection with subsequent symptoms”) and consistently framing analyses as comparing asymptomatic vs symptom-onset–proximate samples, not confirmed pre-UTI states. Where possible, briefly indicate whether these episodes led to clinical encounters or antibiotic treatment.

2. Urotype definition and stability

Urotypes are defined as a predominant genus ≥50% of reads, but stability is described mostly qualitatively. It would help to:

• Explicitly define “no-predominant” urotypes and how Escherichia/Shigella were combined.

• Provide simple descriptive summaries: proportion of participants whose urotype remained the same across two asymptomatic samples; frequency of transitions between major urotypes; how often transitions occurred in pairs that included symptom-onset–proximate samples.

3. Presentation of diversity and Bray–Curtis results

The alpha diversity and Bray–Curtis results would benefit from more concrete summary statistics in text or a concise table (e.g., medians and IQRs by sex and symptom category for each time pair). This would make patterns like lower Bray–Curtis distances between asymptomatic pairs in females and the bimodal pattern in males easier to appreciate, without adding formal hypothesis testing.

4. Relationship to the companion “urinary markers” paper

Because Table 1 and Figure 1 are reprinted and central to interpreting sample groups, please ensure this paper is self-contained:

• Briefly summarize how Groups 1–5 align with common clinical UTI definitions.

• State clearly what is unique to this manuscript (16S-based urobiome variability) versus what is covered in the companion work (urinalysis, culture, biomarkers).

You might consider moving Figure 1 to the supplement and retaining a short textual summary of group distributions by sex.

Policy and clinical-practice implications

The Discussion already connects the findings to diagnostic and antibiotic stewardship, and this could be strengthened with a few explicit statements about implications for policy and practice:

• The authors might consider adding that their findings argue against routine culture or advanced microbiome testing in asymptomatic individuals with NLUTD due to SCI/D, since such testing will frequently identify “abnormal” taxa that actually fall within the normal range of intra individual variability and are unlikely to warrant treatment.

• It would be helpful if the Discussion explicitly linked these results to diagnostic and antibiotic stewardship policies by emphasizing that symptom-based criteria should remain the primary trigger for UTI treatment decisions, with culture and sequencing data interpreted only in the context of each patient’s longitudinal clinical trajectory rather than population-level “healthy” norms.

• The authors might also wish to note that future guideline and research agendas should prioritize longitudinal, patient-level designs that define individualized baselines and actionable thresholds for change, rather than additional cross-sectional sequencing studies that are unlikely to yield clinically usable definitions of “healthy” or “pathogenic” urotypes.

Minor comments

1. Abstract

The abstract is dense and somewhat repetitive. Streamlining repeated phrases and adding one or two specific quantitative descriptors (e.g., proportion of asymptomatic samples with Escherichia/Klebsiella/Enterococcus predominance; typical range of Bray–Curtis values) would make the findings more concrete and concise.

2. Methods

• Clarify the recruitment setting and whether participants were consecutively approached or recruited by convenience.

• Indicate how missing USQNB-IC questionnaires were handled (e.g., rescheduling vs exclusion).

• Specify whether non-antibiotic urinary prophylaxis (such as methenamine) was allowed.

3. Figures and tables

• Figure 2 is informative but visually dense. Consider simplifying the main-text version (e.g., combining low-frequency genera into “other”) and providing a full version in the supplement.

• Table 2 could be complemented by a short table summarizing numbers of samples per participant (1, 2, 3, 4) and counts of asymptomatic versus symptom-onset–proximate samples.

4. Terminology and consistency

Ensure consistent use of “NLUTD due to SCI/D” and of labels for “asymptomatic” vs “symptom-onset–proximate” samples in text and figure legends. A brief definition of “urotype” and “predominance category” early in Methods would help readers follow later results.

Reviewer #2: The manuscript addresses an important and clinically relevant topic regarding longitudinal variability of the urobiome in individuals with SCI/D using intermittent catheterization. The longitudinal design and repeated sampling strategy are strengths of the study and provide valuable insight into intra-individual microbial variability.

The methodology is generally appropriate and clearly described, and the conclusions are mostly supported by the presented findings. The manuscript is well written and logically organized.

I have a few minor suggestions for improvement:

1. The authors may consider further clarifying the rationale for defining “pre-symptomatic” samples, especially since no participant developed clinically confirmed UTI.

2. Some figures, particularly the stacked bar plots, are visually dense and may benefit from improved readability or simplified presentation.

3. The discussion could briefly address the potential influence of catheterization frequency, hydration status, or dietary variation on temporal microbiome variability.

4. The Data Availability Statement should clarify whether anonymized sequencing data will be publicly accessible immediately upon publication and include repository details if available.

Overall, this is a thoughtful and technically sound study that contributes important evidence regarding interpretation of urobiome findings in NLUTD populations.

**********

what does this mean?). If published, this will include your full peer review and any attached files.

If you choose “no”, your identity will remain anonymous but your review may still be made public.

**Do you want your identity to be public for this peer review?**  For information about this choice, including consent withdrawal, please see our Privacy Policy

Reviewer #1: No

Reviewer #2: **Yes:**  Dr. Mohammed Faris Abdulghani

**********

[NOTE: If reviewer comments were submitted as an attachment file, they will be attached to this email and accessible via the submission site. Please log into your account, locate the manuscript record, and check for the action link "View Attachments". If this link does not appear, there are no attachment files.]

To ensure your figures meet our technical requirements, please review our figure guidelines: https://journals.plos.org/plosone/s/figures

You may also use PLOS’s free figure tool, NAAS, to help you prepare publication quality figures: https://journals.plos.org/plosone/s/figures#loc-tools-for-figure-preparation.

NAAS will assess whether your figures meet our technical requirements by comparing each figure against our figure specifications.

# Author comment: Author response to Decision Letter 1

**DOI:** 10.1371/journal.pone.0356391.r002

6 Jul 2026

See Responses to Reviewers (attached)

Attachment Submitted filename: responses to reviewers A1P2 16june2026.docx

# Peer review: Decision Letter 1

**DOI:** 10.1371/journal.pone.0356391.r003

**Authors:** Zongxin Ling

31 Jul 2026

Dear Dr. Tractenberg,

Thank you for submitting your manuscript to PLOS ONE. After careful consideration, we feel that it has merit but does not fully meet PLOS ONE’s publication criteria as it currently stands. Therefore, we invite you to submit a revised version of the manuscript that addresses the points raised during the review process.

Please submit your revised manuscript by Sep 14 2026 11:59PM. If you will need more time than this to complete your revisions, please reply to this message or contact the journal office at plosone@plos.org. When you're ready to submit your revision, log on to https://www.editorialmanager.com/pone/ and select the 'Submissions Needing Revision' folder to locate your manuscript file.

- A letter that responds to each point raised by the academic editor and reviewer(s). You should upload this letter as a separate file labeled 'Response to Reviewers'.
- A marked-up copy of your manuscript that highlights changes made to the original version. You should upload this as a separate file labeled 'Revised Manuscript with Track Changes'.
- An unmarked version of your revised paper without tracked changes. You should upload this as a separate file labeled 'Manuscript'.

If you would like to make changes to your financial disclosure, please include your updated statement in your cover letter. Guidelines for resubmitting your figure files are available below the reviewer comments at the end of this letter.

If applicable, we recommend that you deposit your laboratory protocols in protocols.io to enhance the reproducibility of your results. Protocols.io assigns your protocol its own identifier (DOI) so that it can be cited independently in the future. For instructions see: https://journals.plos.org/plosone/s/submission-guidelines#loc-laboratory-protocols. Additionally, PLOS ONE offers an option for publishing peer-reviewed Lab Protocol articles, which describe protocols hosted on protocols.io. Read more information on sharing protocols at https://plos.org/protocols?utm_medium=editorial-email&utm_source=authorletters&utm_campaign=protocols.

As the corresponding author, your ORCID iD is verified in the submission system and will appear in the published article. PLOS supports the use of ORCID, and we encourage all coauthors to register for an ORCID iD and use it as well. Please encourage your coauthors to verify their ORCID iD within the submission system before final acceptance, as unverified ORCID iDs will not appear in the published article. *Only*  the individual author can complete the verification step; PLOS staff *cannot*  verify ORCID iDs on behalf of authors.

We look forward to receiving your revised manuscript.

Kind regards,

Zongxin Ling

Academic Editor

PLOS One

Journal Requirements:

1. If the reviewer comments include a recommendation to cite specific previously published works, please review and evaluate these publications to determine whether they are relevant and should be cited. There is no requirement to cite these works unless the editor has indicated otherwise.

2. Please review your reference list to ensure that it is complete and correct. If you have cited papers that have been retracted, please include the rationale for doing so in the manuscript text, or remove these references and replace them with relevant current references. Any changes to the reference list should be mentioned in the rebuttal letter that accompanies your revised manuscript. If you need to cite a retracted article, indicate the article’s retracted status in the References list and also include a citation and full reference for the retraction notice.

[Note: HTML markup is below. Please do not edit.]

Reviewer's Responses to Questions

**Comments to the Author**

Reviewer #1: All comments have been addressed

Reviewer #2: All comments have been addressed

**********

2. Is the manuscript technically sound, and do the data support the conclusions?

Reviewer #1: Yes

Reviewer #2: Partly

**********

3. Has the statistical analysis been performed appropriately and rigorously?

Reviewer #1: Yes

Reviewer #2: Yes

**********

4. Have the authors made all data underlying the findings in their manuscript fully available?

The PLOS Data policy

Reviewer #1: No

Reviewer #2: Yes

**********

5. Is the manuscript presented in an intelligible fashion and written in standard English?

Reviewer #1: Yes

Reviewer #2: Yes

**********

Reviewer #1: The authors addressed most major comments thoroughly—urotype concordance, descriptive statistics, and clinical stewardship implications are now substantially strengthened.

Two substantive variances remain:

Terminology ("presymptomatic" vs. "asymptomatic at collection with subsequent symptoms"): Clarified but not resolved. The term persists and may still prompt concern about clinical inference.

Groups 1–5 alignment with UTI definitions: Declined.

Reviewer #2: Thank you for the opportunity to review this revised manuscript entitled "Variation in Urobiome Composition Over Time in Asymptomatic Individuals with Spinal Cord Injury and Disease Using Intermittent Catheterization." The study addresses an important clinical problem by investigating longitudinal variability of the urinary microbiome in individuals with neurogenic lower urinary tract dysfunction (NLUTD). The longitudinal design, repeated sampling strategy, and emphasis on distinguishing normal microbial variation from clinically meaningful changes represent notable strengths. The manuscript is well written and provides valuable data that may contribute to improving microbiome-informed diagnosis of urinary tract infection in this population. Nevertheless, several issues should be addressed before the manuscript is suitable for publication.

Major Comments

1. Clarify the novelty of the study. Although the manuscript emphasizes the limitations of cross-sectional microbiome studies, the introduction should more explicitly state how this study advances current knowledge beyond previous longitudinal urobiome investigations. A concise statement highlighting the unique contribution and clinical implications would strengthen the rationale.

2. Sample size justification. The manuscript includes 94 participants; however, no sample size calculation or justification is provided. Even for a descriptive longitudinal study, the authors should explain how the sample size was determined and discuss its adequacy for addressing the study objectives.

3. Handling of repeated measurements. While the study is primarily descriptive, participants contributed varying numbers of urine samples over time. The manuscript should explain how unequal numbers of repeated observations were handled and discuss whether this may have influenced the reported variability.

4. Potential confounding factors. Several factors known to influence the urinary microbiome—including age, sex, underlying neurological diagnosis, duration of disease, catheterization frequency, diet, hydration, bowel management, and prior antibiotic exposure—are not explored. At minimum, the limitations section should acknowledge these potential confounders and discuss their possible impact on microbial variability.

5. Statistical methodology. The exclusive reliance on descriptive statistics limits the strength of the conclusions. Consider including confidence intervals or additional exploratory longitudinal analyses to quantify the stability of microbial composition. Even if inferential analyses are intentionally avoided, the rationale for this decision should be explained more clearly.

6. Interpretation of the findings. Some conclusions regarding the limitations of cross-sectional studies appear stronger than the data directly support. The discussion should be revised to avoid overgeneralization and acknowledge that the findings are specific to adults with NLUTD using intermittent catheterization.

7. Clinical implications. The discussion would benefit from a more detailed explanation of how these findings could influence future diagnostic algorithms for urinary tract infection, antimicrobial stewardship, and personalized microbiome-based clinical decision-making.

8. Limitations. The limitations section should be expanded to include the single-center nature of the cohort, limited generalizability to other bladder-management methods, reliance on 16S rRNA sequencing rather than shotgun metagenomics, inability to evaluate microbial function, and the absence of host immune or inflammatory biomarkers.

Further comments

1. The abstract is somewhat lengthy and repetitive. Consider shortening the concluding sentences to improve readability.

2. Several paragraphs in the Introduction are considerably long and could be divided into shorter sections to improve flow.

3. The Methods section contains duplicated descriptions of DNA extraction/genome extraction. These sections should be consolidated to avoid redundancy.

4. Please define all abbreviations at their first appearance and ensure consistent use throughout the manuscript.

5. Figure legends should be expanded to allow readers to understand the figures without referring extensively to the main text.

6. Table 2 would be more informative if percentages were reported alongside frequencies where appropriate.

7. Please ensure that all cited references are up to date and formatted consistently according to journal style.

8. Carefully proofread the manuscript for minor grammatical inconsistencies and formatting issues, particularly spacing around citations and punctuation.

This manuscript addresses an important and clinically relevant topic and has several methodological strengths, particularly its prospective longitudinal design and rigorous symptom monitoring. The findings provide valuable insight into temporal variability of the urobiome in individuals with SCI/D and have potential implications for future microbiome-informed diagnostics. However, clarification of the methodology, expansion of the discussion, and a more balanced interpretation of the findings are necessary before publication.

**********

what does this mean?). If published, this will include your full peer review and any attached files.

If you choose “no”, your identity will remain anonymous but your review may still be made public.

**Do you want your identity to be public for this peer review?**  For information about this choice, including consent withdrawal, please see our Privacy Policy

Reviewer #1: No

Reviewer #2: **Yes:**  Dr. Mohammed Faris Abdulghani

**********

[NOTE: If reviewer comments were submitted as an attachment file, they will be attached to this email and accessible via the submission site. Please log into your account, locate the manuscript record, and check for the action link "View Attachments". If this link does not appear, there are no attachment files.]

To ensure your figures meet our technical requirements, please review our figure guidelines: https://journals.plos.org/plosone/s/figures

You may also use PLOS’s free figure tool, NAAS, to help you prepare publication quality figures: https://journals.plos.org/plosone/s/figures#loc-tools-for-figure-preparation.

NAAS will assess whether your figures meet our technical requirements by comparing each figure against our figure specifications.

# Author comment: Author response to Decision Letter 2

**DOI:** 10.1371/journal.pone.0356391.r004

2 Aug 2026

Responses to reviewers on R2 are uploaded as a separate document.

Attachment Submitted filename: R2 Responses to Reviewers.docx

# Editor report: Decision Letter 2

**DOI:** 10.1371/journal.pone.0356391.r005

**Authors:** Zongxin Ling

3 Aug 2026

Variation in urobiome composition over time in asymptomatic individuals with spinal cord injury and disease using intermittent catheterization

PONE-D-26-11433R2

Dear Dr. Tractenberg,

We’re pleased to inform you that your manuscript has been judged scientifically suitable for publication and will be formally accepted for publication once it meets all outstanding technical requirements.

Within one week, you’ll receive an e-mail detailing the required amendments. When these have been addressed, you’ll receive a formal acceptance letter and your manuscript will be scheduled for publication.

An invoice will be generated when your article is formally accepted. Please note, if your institution has a publishing partnership with PLOS and your article meets the relevant criteria, all or part of your publication costs will be covered. Please make sure your user information is up-to-date by logging into Editorial Manager at Editorial Manager® and clicking the ‘Update My Information' link at the top of the page. For questions related to billing, please contact billing support.

If your institution or institutions have a press office, please notify them about your upcoming paper to help maximize its impact. If they’ll be preparing press materials, please inform our press team as soon as possible -- no later than 48 hours after receiving the formal acceptance. Your manuscript will remain under strict press embargo until 2 pm Eastern Time on the date of publication. For more information, please contact onepress@plos.org.

Kind regards,

Zongxin Ling

Academic Editor

PLOS One

Additional Editor Comments (optional):

Reviewers' comments:

# Editor report: Acceptance letter

**DOI:** 10.1371/journal.pone.0356391.r006

**Authors:** Zongxin Ling

PONE-D-26-11433R2

PLOS One

Dear Dr. Tractenberg,

I'm pleased to inform you that your manuscript has been deemed suitable for publication in PLOS One. Congratulations! Your manuscript is now being handed over to our production team.

At this stage, our production department will prepare your paper for publication. This includes ensuring the following:

* All references, tables, and figures are properly cited

* All relevant supporting information is included in the manuscript submission,

* There are no issues that prevent the paper from being properly typeset

You will receive further instructions from the production team, including instructions on how to review your proof when it is ready. Please keep in mind that we are working through a large volume of accepted articles, so please give us a few days to review your paper and let you know the next and final steps.

Lastly, if your institution or institutions have a press office, please let them know about your upcoming paper now to help maximize its impact. If they'll be preparing press materials, please inform our press team within the next 48 hours. Your manuscript will remain under strict press embargo until 2 pm Eastern Time on the date of publication. For more information, please contact onepress@plos.org.

You will receive an invoice from PLOS for your publication fee after your manuscript has reached the completed accept phase. If you receive an email requesting payment before acceptance or for any other service, this may be a phishing scheme. Learn how to identify phishing emails and protect your accounts at https://explore.plos.org/phishing.

If we can help with anything else, please email us at customercare@plos.org.

Thank you for submitting your work to PLOS One and supporting open access.

Kind regards,

PLOS One Editorial Office Staff

on behalf of

Dr. Zongxin Ling

Academic Editor

PLOS One
